# Development and Validation of a Hybrid Machine Learning Model to Predict Lung Transplant Outcomes

**DOI:** 10.1001/jamanetworkopen.2025.45369

**Published:** 2025-11-25

**Authors:** Gaurav Sharma, Vineet Kumar Kamal, Srinivas Bollineni, Irina Timofte, Jonathan D. Plasencia, Srdjan Lesaja, Vaidehi Kaza, Suresh Keshavamurthy, John Murala, Matthias Peltz, Michael E. Jessen

**Affiliations:** 1 Department of Cardiovascular and Thoracic Surgery, University of Texas Southwestern Medical Center, Dallas; 2 Advanced Imaging Research Center, University of Texas Southwestern Medical Center, Dallas; 3 Department of Biomedical Engineering, University of Texas Southwestern Medical Center, Dallas; 4 Department of Biostatistics, All India Institute of Medical Sciences, Kalyani, India; 5 Division of Pulmonary and Critical Care Medicine, Department of Internal Medicine, University of Texas Southwestern Medical Center, Dallas; 6 Department of Research, United Network for Organ Sharing, Richmond, Virginia

## Abstract

**Importance:**

Long-term survival after a lung transplant remains highly variable, and existing risk stratification tools have limited accuracy, clinical utility, and interpretability.

**Objective:**

To develop, validate, and assess the clinical utility of an interpretable hybrid machine learning model using United Network for Organ Sharing data to predict time to death or retransplant at 1, 5, and 10 years after a lung transplant.

**Design, Setting, and Participants:**

This prognostic study used data from a United Network for Organ Sharing–Organ Procurement and Transplantation Network cohort that underwent lung transplants between October 16, 1987, and March 26, 2025. The study included 51 933 adult patients (aged ≥18 years) undergoing their first lung transplant in the US. The data were temporally split into a development cohort (1987-2014; n = 26 682) and a testing cohort (2015-2025; n = 25 251). The development cohort was divided into a training set (n = 24 014) and validation set (n = 2668) for model selection and hyperparameter tuning.

**Main Outcomes and Measures:**

The outcome was the time to death or retransplant. The model was developed using the AutoScore-Survival framework, which uses a random survival forest for variable selection and Cox proportional hazards regression for scoring. Performance was assessed by discrimination (a time-dependent area under the curve [AUC], the Harrell C-index, and integrated AUC [iAUC]), calibration (plots, slope, observed-to-expected event ratio, and Brier score), and clinical utility (decision curve analysis).

**Results:**

Among 51 933 recipients (median age, 59 years [5th-95th percentile range, 27-71 years]; 57.6% men), the median follow-up was 8.97 years (95% CI, 8.93-8.99 years), and 31 865 (61.4%) experienced an event. Nine predictors were selected for the final model: length of hospital stay, recipient age, single vs double transplant, posttransplant ventilation support, prior cardiac surgery, creatinine level at transplant, functional status, total bilirubin level, and donor age. In the unseen testing set, the model showed moderate discrimination with an iAUC of 0.61 (95% CI, 0.60-0.63) and a C-index of 0.64 (95% CI, 0.63-0.64). The time-dependent AUC was 0.61 (95% CI, 0.52-0.70) at 1 year, 0.59 (95% CI, 0.53-0.65) at 5 years, and 0.72 (95% CI, 0.55-0.85) at 10 years. The model was well calibrated, and the decision curve analysis demonstrated a consistent net benefit across threshold probabilities.

**Conclusions and Relevance:**

In this large prognostic study, the interpretable hybrid model provided practical, personalized risk stratification for lung transplant outcomes. With moderate discrimination, good calibration, and clear clinical utility, the model supports shared decision-making and is accessible via a web-based calculator.

## Key Points

**Question:**

Can an interpretable hybrid machine learning model predict 1-, 5-, and 10-year risk of death or retransplant after a lung transplant?

**Findings:**

In this prognostic study using a UNOS-OPTN cohort of 51 933 adults undergoing a first lung transplant, a 9-variable AutoScore-Survival model showed moderate discrimination (integrated area under the curve, 0.61; C-index, 0.64), good calibration, and net clinical benefit on decision-curve analysis in the testing cohort across time horizons.

**Meaning:**

These findings suggest that this interpretable, web-accessible risk calculator may support individualized posttransplant risk stratification, patient counseling, and shared decision-making in clinical practice.

## Teaser

This prognostic study uses United Network for Organ Sharing data to develop, validate, and assess the clinical utility of an interpretable hybrid machine learning model to predict time to death or retransplant at 1, 5, and 10 years after a lung transplant.

## Introduction

A lung transplant remains the only lifesaving intervention for patients with end-stage pulmonary diseases. Despite significant advances in surgical techniques and immunosuppressive therapies, posttransplant survival remains highly variable.^1,2^ Traditional risk models, often based on Cox proportional hazards regression, have provided valuable insights but often fail to capture the complex interactions among donor, recipient, and perioperative factors.^3^ Moreover, the increasing volume of high-dimensional clinical data supports a role for the integration of machine learning methods to improve predictive accuracy while maintaining interpretability.^4^ Although many machine learning models achieve a superior predictive performance, their black-box nature often limits clinical adoption, as clinicians require transparency to understand the association of individual variables with a patient’s risk profile.^5,6,7^ Scores such as the historical Lung Allocation Score and the current Composite Allocation Score are essential for pretransplant organ allocation, but their primary function is prioritizing waiting list candidates, not long-term postdischarge prognostication. However, a critical gap remains; there is no widely adopted, parsimonious, or interpretable postdischarge risk score designed for long-term patient monitoring that has been rigorously validated on a contemporary patient cohort. This study aimed to fill this gap by developing and temporally validating such a tool.^8,9^

The AutoScore-Survival framework represents a novel approach to this challenge.^9^ This hybrid method systematically integrates the variable ranking power of random survival forests (a robust machine learning technique) with the inherent interpretability of Cox proportional hazards regression to generate a simple, point-based clinical scoring system.^10^ By automating the creation of an integer-based score, this framework produces a statistically sound and practical tool for bedside use, addressing a key barrier that has limited the clinical effect of more complex models.^11,12^ Although studied in other clinical contexts, this method has not yet been applied to lung transplants.^13^ Existing posttransplant models either focus on short-term graft dysfunction or sacrifice interpretability for marginal gains in discrimination.^14,15,16^ In this study, we use the United Network for Organ Sharing (UNOS)–Organ Procurement and Transplantation Network (OPTN) database to derive, validate, and assess the clinical utility of a time-to-event risk prediction model for recipients of a lung transplant. We hypothesize that an interpretable, parsimonious risk score, using a limited number of key variables, can provide clinicians with a powerful yet user-friendly tool to predict 1-, 5-, and 10-year posttransplant outcomes, thereby enabling tailored postoperative management and resource allocation.^10,17^

## Methods

### Study Design and Population

A prognostic study was conducted using data on a cohort from the UNOS-OPTN registry, which captures detailed information on recipients of a lung transplant from across the US. This report followed the Transparent Reporting of a Multivariable Prediction Model for Individual Prognosis or Diagnosis + Artificial Intelligence (TRIPOD+AI) reporting guidelines. This study was conducted using deidentified secondary data from the UNOS registry. The study protocol was reviewed by the institutional review board at University of Texas Southwestern Medical Center and was determined to be exempt from the requirement for informed consent because no direct patient or public involvement was included in the research.

**Figure 1.**
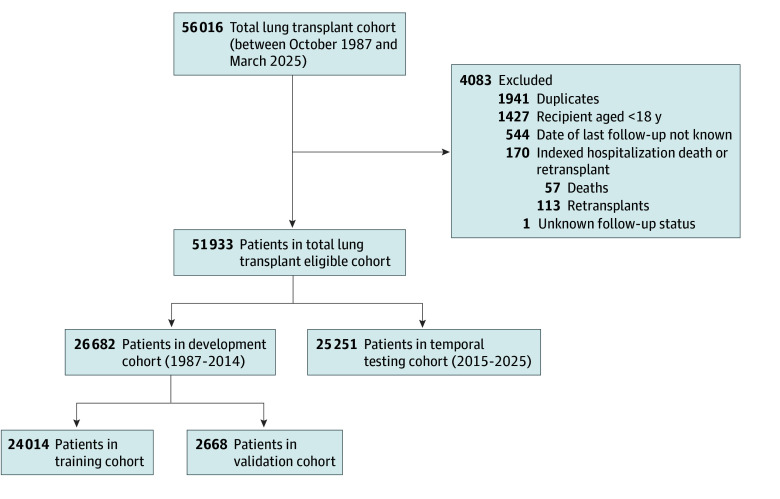
Patient Selection and Study Cohort Allocation Inclusion and exclusion criteria applied to the United Network for Organ Sharing database to identify the final study cohort of 51 933 recipients of a lung transplant. Patients were temporally assigned to development (1987-2014) and testing (2015-2025) cohorts, with the development cohort randomly split into training (90%) and validation (10%) sets.

As illustrated in the study flowchart (Figure 1), this study included all adult patients (aged ≥18 years) who underwent a lung transplant between October 16, 1987, and March 26, 2025. The final cohort included 51 933 patients. The cohort was temporally divided into a development set (those who received a transplant from 1987 to 2014; n = 26 682) and a testing set (those who received a transplant from 2015 to 2025; n = 25 251). This temporal validation approach provides a robust assessment of the model’s generalizability to a contemporary patient population, simulating clinical prospective application. The development set was then randomly split into a training cohort (90% [n = 24 014]) and a validation cohort (10% [n = 2668]). The purpose of each cohort is described in the eMethods in Supplement 1.

### Sample Size and Missing Data

The sample size was determined a priori (n = 28 680) for model development, as detailed in the eMethods in Supplement 1. Missing values for some variables were preserved as an unknown or not reported category (a category in UNOS) and included in the analysis, an approach that acknowledges that the reason for missingness can carry prognostic information.^18,19,20^ Missing data handling procedures are described in the eMethods in Supplement 1.

### Outcome Definition and Candidate Variables

The primary outcome was defined as a time to event, representing the duration from lung transplant to either death or retransplant. Survival outcomes were assessed at 1, 5, and 10 years after the transplant. We identified 61 candidate variables based on an extensive review of the literature and clinical expertise. These variables encompassed recipient demographic characteristics (eg, age, gender, race and ethnicity [Black, Hispanic, White, and other (American Indian or Alaska Native, Asian, Native Hawaiian or Other Pacific Islander, multiracial, and unknown)], body mass index, and functional status), donor characteristics (eg, age and smoking history), and transplant-related factors (eg, ischemic time and type of lung transplant inotropic support). Race and ethnicity were reported as recorded in the UNOS-OPTN registry, where they are collected based on self-report or information from next of kin according to predefined categories. The comprehensive list of 61 candidate variables and their definitions is provided in eTable 1 in Supplement 1.

### Statistical Analysis

#### Model Development and Performance Evaluation

The model was developed using the AutoScore-Survival framework, a multistep algorithm integrating a random survival forest for variable selection and Cox proportional hazards regression for score generation. A parsimony plot, generated using the validation cohort, was used to select the optimal number of variables by balancing model complexity with predictive accuracy (eFigure 2 in Supplement 1). The final model’s performance was evaluated in the testing dataset using a time-dependent area under the curve (AUC), the Harrell C-index, and an integrated AUC (iAUC).^3^ The model development process is detailed in the eMethods in Supplement 1.

#### Calibration

Calibration, the agreement between predicted and observed outcomes, was assessed with calibration plots, the calibration slope, the observed-to-expected ratio, and the Brier score. Bootstrapping with 200 resamples was performed to generate 95% CIs for these performance measures. Calibration plots compared predicted and observed survival across risk deciles. Clinical utility was evaluated using decision curve analysis in the testing cohort. Finally, a web-based risk calculator was developed using the Shiny package in R, version 4.4.1 (R Project for Statistical Computing), allowing real-time prediction of death or retransplant probabilities for recipients of a lung transplant based on the AutoScore-derived risk model. Details about other statistical analyses and the software used are in the eMethods in Supplement 1.

## Results

### Patient Characteristics and Cohort Description

Among 51 933 adult recipients of a lung transplant included in the final cohort (Figure 1), the median age was 59 years (5th-95th percentile range, 27-71 years), 29 905 (57.6%) were men, 22 028 (42.4%) were women, 4435 (8.5%) were Black, 4067 (7.8%) were Hispanic, 41 904 (80.7%) were White, and 1527 (2.9%) were other race or ethnicity (eTable 2 in Supplement 1). The distribution of baseline characteristics was comparable across the training, validation, and testing cohorts for most of the variables. Frequencies of outcome, median follow-up, and event-free survival time are given in eTable 3 in Supplement 1. For the entire cohort, the overall event rate was 61.4% (n = 31 865), the median follow-up time was 8.97 years (95% CI, 8.93-8.99 years), and the median event-free survival was 5.79 years (95% CI, 5.71-5.88 years). The overall Kaplan-Meier survival curve is shown in eFigure 1 in Supplement 1. The results of univariable and multivariable Cox proportional hazards regression for all candidate variables are presented in eTable 4 in Supplement 1.

### Variable Selection and Model Development

**Figure 2.**
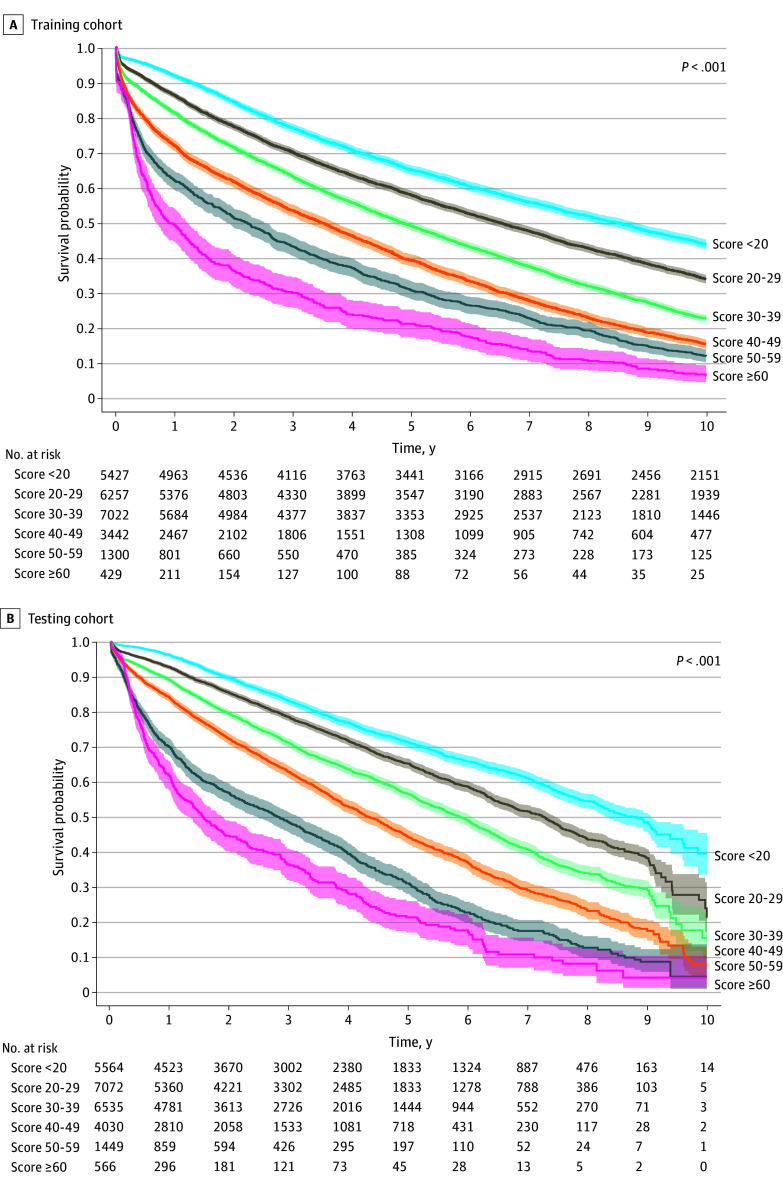
Kaplan-Meier Estimates of Survival by Risk Score Kaplan-Meier survival curves show overall survival stratified by risk score categories in the (A) training cohort and (B) testing cohort. Higher risk scores were associated with significantly lower survival probabilities (log-rank *P* < .001).

Using the AutoScore-Survival framework for the model development in the training cohort (n = 24 014), in the first step, a weight was assigned to each of the 61 candidate variables, and the variables were ranked according to their importance using a random survival forest, with the final number determined via parsimony plot analysis (ie, model performance vs complexity) based on the validation cohort (n = 2668) (eFigure 2 in Supplement 1). The optimal balance between model performance (iAUC) and complexity was achieved with 9 variables: length of hospital stay, recipient age, type of transplant (single vs double), posttransplant ventilation support, prior cardiac surgery, creatinine level of recipient at transplant, functional status at the time of transplant, total bilirubin level, and donor age, resulting in a 9-variable AutoScore-Survival–derived scoring system, as shown in Table 1. In our scoring system model, based on training cohort data, a hospital lengths of stay of less than 6 days and 70 or more days received the highest scores (<6 days, 24; ≥70 days, 23), highlighting that shorter and prolonged hospital stays were associated with increased risk of death or retransplant after the first lung transplant. This variable was followed by recipient age of 68 years or older, which contributed 20 points, indicating the significant association of older age. Table 1 and Figure 2A illustrate the association between risk scores and predicted survival probabilities in the training cohort. For example, patients with risk scores less than 20 demonstrated the highest survival probability, whereas those with scores of 60 or more had the lowest survival probability, indicating a strong association between increasing risk scores and poorer outcomes. As shown in Figure 2B, the time-to-event score effectively stratifies patients into distinct risk groups in the testing cohort as well, with significantly different survival probabilities across risk categories, where higher risk scores correlated with progressively worse survival, validating the model’s accuracy. The final model was obtained via recalibration-in-the-large on the 2015 to 2025 cohort, fitting a Cox proportional hazards regression model with the development linear predictor as an offset to reestimate only the baseline hazard (eMethods in Supplement 1).

**Table 1.** Risk Score for Predicting Death or Retransplant After a Lung Transplant

Variable or interval	Score points^a^	HR (95% CI)
Length of hospital stay, d		
<6	24	1.69 (1.56-1.84)
6-9	0	1 [Reference]
10-30	3	1.10 (1.06-1.15)
31-69	10	1.33 (1.27-1.41)
≥70	23	1.92 (1.78-2.06)
Recipient age, y		
<25	11	1.37 (1.27-1.47)
25-41	0	1 [Reference]
42-62	7	1.22 (1.17-1.28)
63-67	16	1.59 (1.50-1.68)
≥68	20	1.80 (1.68-1.93)
Single or double lung transplant		
Double	0	1 [Reference]
Single	12	1.42 (1.38-1.47)
Posttransplant ventilation support		
No or VS for ≤48 h or unknown or not reported	0	1 [Reference]
VS for >48 h but <5 d	2	1.07 (1.01-1.12)
VS for ≥5 d	10	1.33 (1.26-1.40)
Prior cardiac surgery		
No	0	1 [Reference]
Yes or unknown	8	1.25 (1.21-1.30)
Creatinine level for recipient at transplant, mg/dL		
<1.00	0	1 [Reference]
1.00-1.29	2	1.05 (1.02-1.09)
≥1.30	7	1.20 (1.13-1.29)
Functional status at time of transplant		
Fully active	0	1 [Reference]
Restricted activities or need assistance	4	1.10 (1.05-1.15)
Severely disabled	7	1.21 (1.14-1.29)
Completely disabled	10	1.32 (1.23-1.41)
Not applicable, unknown, or not reported	8	1.26 (1.19-1.33)
Total bilirubin, mg/dL		
<1.4	0	1 [Reference]
≥1.4	4	1.13 (1.06-1.21)
Donor age, y		
<47	0	1 [Reference]
47-56	3	1.08 (1.04-1.12)
≥57	5	1.13 (1.07-1.20)

Abbreviations: HR, hazard ratio; VS, ventilator stay.

SI conversion factors: To convert creatinine to micromoles per liter, multiply by 88.4; and bilirubin to micromoles per liter, multiply by 17.104.

^a^ The 9-variable AutoScore-Survival–derived scoring system is used to predict time-to-event outcomes in recipients of a lung transplant. Variables included in the final model were length of hospital stay, recipient age, single vs double lung transplant, posttransplant ventilation support, prior cardiac surgery, creatinine level for recipient at transplant, functional status at the time of transplant, total bilirubin level, and donor age. Scores are assigned based on categorized risk factors. In addition, HRs with 95% CIs from the adjusted multivariable analysis, incorporating all 9 variables, are provided for each risk category.

### Performance Measures and Clinical Utility

**Figure 3.**
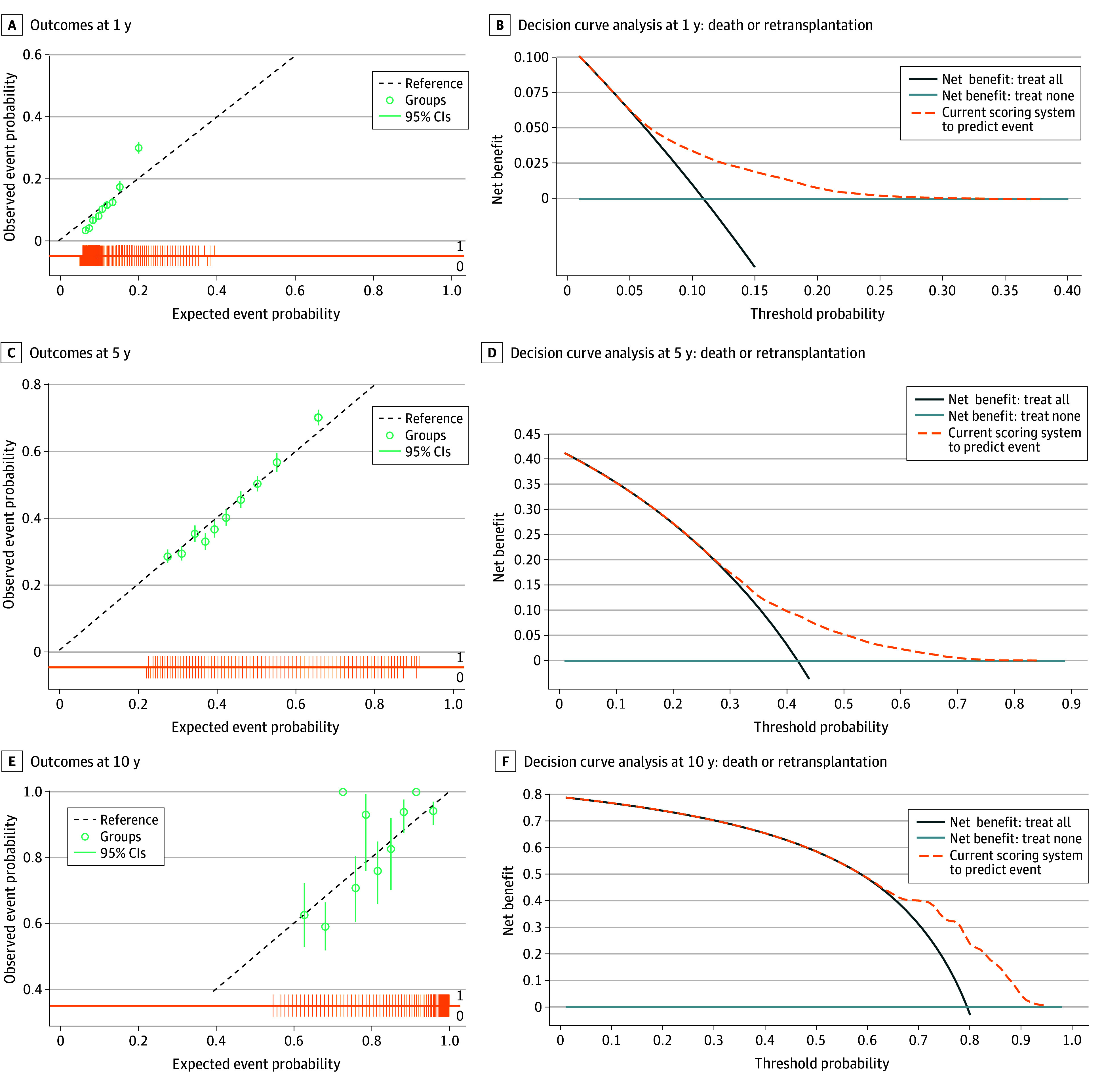
Model Calibration and Clinical Utility in the Testing Cohort Panels display model calibration plots (A, C, and E) and their corresponding decision curve analyses (B, D, and F) for outcomes at 1 year (A and B), 5 years (C and D), and 10 years (E and F), respectively. The alignment of groups with the reference line in panels A, C, and E indicates calibration. In panels B, D, and F, the net benefit of the scoring system (dashed line) is compared against treating all patients or no patients. Calibration plots compared predicted vs observed event probabilities. The circles indicate deciles of predicted risk, vertical lines in the circles indicate 95% CIs, and their alignment with the dashed diagonal line reference line indicates the degree of calibration. Decision curve analyses evaluate the model’s clinical utility. The net benefit of using the current scoring system is compared with the default strategies of intervening for all patients (treat all) or for no patients (treat none; horizontal line at zero). A higher curve indicates greater clinical value across the range of threshold probabilities.

The performance of the model in the training and testing cohorts is summarized in Table 2. In the unseen testing cohort, the model demonstrated moderate discrimination with an iAUC of 0.61 (95% CI, 0.60-0.63) and a C-index of 0.64 (95% CI, 0.63-0.64). The model was well recalibrated, as shown in the calibration plots for the testing cohort, where predicted probabilities closely aligned with observed outcomes at 1, 5, and 10 years (Figure 3A). The calibration plots for the training and testing cohorts (without recalibration) are provided in eFigure 3 in Supplement 1. Decision curve analysis in the testing cohort (Figure 3B) using the recalibrated model showed that the scoring system provided a higher net benefit than both the treat-all and treat-none strategies across a wide range of clinically relevant threshold probabilities. This finding suggests that using the model to guide clinical decisions is superior to assuming all patients are at high risk or low risk, confirming its clinical utility.

**Table 2.** Model Performance in Training and Testing Cohorts

Performance measure^a^	Cohort	
	Training (n = 24 014)	Testing (n = 25 251)
Discrimination ability		
iAUC (95% CI)	0.66 (0.65-0.67)	0.61 (0.60-0.63)
C-index (95% CI)	0.60 (0.60-0.61)	0.64 (0.63-0.64)
Time-dependent AUC at 1 y (95% CI)	0.70 (0.69-0.71)	0.61 (0.52-0.70)
Time-dependent AUC at 5 y (95% CI)	0.63 (0.62-0.63)	0.59 (0.53-0.65)
Time-dependent AUC at 10 y (95% CI)	0.66 (0.65-0.67)	0.72 (0.55-0.85)
*R*^2^ (95% CI), %^b^	7.2 (6.6-7.9)	10.7 (9.7-11.8)
Calibration ability		
Calibration slope (95% CI)	1 (0.96-1.04)	1.22 (1.17-1.28)^c^
Observed-to-expected ratio		
At 1 y	0.99	0.99
At 5 y	0.99	0.99
At 10 y	1.00	1.00
Brier score		
At 1 y	3.8 × 10^−6^	4.7 × 10^−7^
At 5 y	9.9 × 10^−6^	2.8 × 10^−5^
At 10 y	1.0 × 10^−6^	8.1 × 10^−6^

Abbreviations: AUC, area under curve; C-index, Harrell concordance index; iAUC, integrated AUC by all time points.

^a^ Performance measures are rounded up to 2 decimals. Higher iAUC and C-index values indicate better discrimination, while observed-to-expected ratios close to 1 suggest accurate calibration. All testing cohort metrics are after recalibration, except for the calibration slope in the testing cohort.

^b^ D-method.

^c^ Without recalibration.

### Model Performance in Subgroups

Model performance was evaluated across different recipient age groups and UNOS geographic regions in the testing cohort. The model maintained almost consistent discrimination across age groups (<40 years, 40-60 years, and >60 years), with iAUC values of 0.66 (95% CI, 0.64-0.70) for those younger than 40 years, 0.66 (95% CI, 0.65-0.68) for those aged 40 to 60 years, and 0.64 (95% CI, 0.62-0.66) for those older than 60 years (eTable 8 in Supplement 1). Time-dependent AUCs at 1, 5, and 10 years also showed consistency across these age bands (eTable 7 in Supplement 1). The calibration plot also remained robust across these age bands except at 10 years (the variation possible due to a relatively smaller sample size) (eFigure 4 in Supplement 1); however, observed-to-expected ratios remained close to 1.0 at 1 year, 5 years, and 10 years (eFigure 5 in Supplement 1). Furthermore, the model demonstrated a stable performance across all 11 UNOS-OPTN transplant regions. The iAUC ranged from 0.61 (95% CI, 0.58-0.66) in region 8 to 0.67 (95% CI, 0.64-0.70) in region 11 (eTable 6 in Supplement 1). Time-dependent AUCs at 1 year, 5 years, and 10 years also showed consistency across regions (eTable 5 in Supplement 1), and calibration plots confirmed regional reliability (eFigures 6 and 7 in Supplement 1), supporting the broad applicability of the model. A publicly accessible, web-based risk calculator providing real-time predictions has been developed^21^ (eFigure 8 in Supplement 1).

## Discussion

In this large prognostic study using data from a national US registry, we developed and temporally validated a parsimonious, 9-variable, point-based risk score to predict the long-term risk of death or retransplant after a lung transplant. The model generated using a hybrid machine learning framework demonstrated moderate discrimination, good calibration, and consistent performance across key clinical subgroups and all US transplant regions. From 61 candidate variables, we identified 9 key predictors: length of hospital stay, recipient age, type of lung transplant (single or double lung transplant), posttransplant ventilation support, prior cardiac surgery, creatinine level at transplant, functional status at transplant, total bilirubin level, and donor age. This model balances predictive accuracy with clinical utility, making it a valuable tool for personalized patient management.

### Comparison With Other Models

The performance and utility of this model should be considered in the context of existing risk stratification tools for lung transplants (eTable 9 in Supplement 1). The most widely used tool, the Lung Allocation Score, was designed for pretransplant prioritization to balance waiting list urgency with posttransplant survival, but its utility for predicting individual long-term outcomes is limited.^22^ In recent years, numerous machine learning models have been developed^14,15,16,23,24^; however, these models often focus on predicting short-term complications, such as primary graft dysfunction, or use black-box algorithms, such as gradient boosting or deep learning, which can achieve higher discrimination at the cost of clinical interpretability. The model in this study occupies a distinct niche; it is a simple, transparent tool for posttransplant risk stratification over the long term. Its moderate discrimination is balanced by excellent calibration and a straightforward, point-based format that facilitates clinical adoption and communication with patients, addressing a key barrier that has limited the clinical effect of more complex models. In addition, most previous models had inadequate sample sizes and suboptimal methods and lacked external validation, calibration, or clinical utility assessment.

### Clinical Interpretation of Predictors

The 9 predictors identified by our model are consistent with established risk factors in the literature.^25^ The selection of both pretransplant (eg, recipient age and functional status) and early posttransplant (eg, length of stay and ventilation support) variables confirms that outcomes are determined by a continuum of factors. The substantial risk score assigned to a very short hospital stay (<6 days) may seem counterintuitive but likely reflects unmeasured confounding; this category may capture patients who experience early, catastrophic events leading to rapid death or transfer to hospice, rather than a smooth recovery. Serum creatinine levels serve as a marker of kidney function, which is increasingly recognized as a factor associated with survival among patients who have received a lung transplant.^26^ The distinction between single and double lung transplants further refines the risk stratification, as outcomes differ significantly between these 2 procedures.^27^ Recipient age and donor age have been repeatedly identified as critical factors associated with posttransplant outcomes, while prolonged hospital stay and need for posttransplant ventilation are indicative of the severity of perioperative complications.

Our hybrid model provides a practical tool for prognostication, using a random survival forest to screen 61 candidate variables and project them onto a Cox proportional hazards regression model. This method retains clinical transparency while exploiting signals that conventional regression would miss.^28^ The integer score conveys each variable’s contribution in familiar hazard ratio units, giving clinicians mechanistic insight and a ready-to-use risk estimate.^14^ Although model discrimination is moderate, per the iAUC and C-index, the benefits of interpretability and ease of use may outweigh the slight decrease in predictive accuracy compared with more complex black-box models.^8^ The web-based risk calculator further enhances the practical applicability of our tool, allowing for dynamic risk assessment and real-time clinical decision support.

### Clinical Implications

An interpretable risk prediction tool can be seamlessly integrated into the electronic health records of transplant centers, facilitating automated risk stratification at the point of care.^29,30^ This can guide tailored posttransplant management strategies, such as close monitoring of patients at high risk or earlier interventions aimed at mitigating complications.^31,32^

This hybrid model achieved a C-index of 0.64 on the unseen temporal dataset, comparable with or better than existing models, and demonstrated good calibration-in-the-large with observed-to-expected ratios near 1.00 with recalibration. This close alignment between predicted and observed event rates, together with the positive net benefit on decision curve analysis across meaningful risk thresholds, indicates that the tool can reliably inform posttransplant monitoring and shared decision-making despite modest discrimination.

### Strengths and Limitations

A key strength of our model is its robust performance across diverse patient populations. The model maintained consistent discrimination and calibration across age groups and all 11 US transplant regions. This robustness, strengthened by rigorous temporal validation, indicates the predictions are not overly sensitive to local practice patterns or patient age and supports the potential of this model for broad clinical applicability (eTables 5-8 and eFigures 4-7 in Supplement 1).

Although the study is grounded in a robust dataset and methodological rigor, several limitations must be recognized in interpreting the findings. The retrospective design, although based on a reliable national registry, may be subject to biases inherent in registry data, such as variability in data quality and completeness. The decision to retain missing values as an informative category, while pragmatic, may introduce confounding that is not entirely accounted for in our model.^33^ Future studies should consider prospective validation and potential external validation using independent datasets to further confirm the performance of the model.^34^ In addition, while the selected 9 predictors offer a balance between simplicity and predictive power, the inclusion of emerging biomarkers and additional clinical variables might enhance discrimination in future iterations of the model. The large score assigned to a length of hospital stay of less than 6 days (24 points) may reflect unmeasured acuity in patients with very short stays, such as early, unrecorded mortality or rapid transfers, warranting further investigation. Furthermore, our development cohort (1987-2014) predates major 2015 US lung allocation system changes, including the transition from the Lung Allocation Score to the Composite Allocation Score. Although the temporal validation cohort (2015-2025) shows model robustness after recalibration, these policy shifts may limit generalizability due to evolving candidate pool characteristics. To avoid incorporation bias, we excluded index-hospitalization deaths and retransplants, when possible, although residual misclassification may bias length of stay associations (eMethods in Supplement 1).

Future research directions include the incorporation of longitudinal data to capture dynamic changes in patient status over time. Integration of donor-derived cell-free DNA or proteomic allograft-injury signatures may improve discrimination without compromising transparency.^19,35^ To ensure clinical applicability, future efforts will focus on external validation across diverse transplant centers and demographic subgroups. Prospective clinical trials and multicenter collaborations will be essential to validate the utility of the risk tool in diverse patient populations and clinical settings.

## Conclusions

We have developed and temporally validated an interpretable, hybrid machine learning model that generates a parsimonious risk score for recipients of a lung transplant. The model incorporates 9 key predictors, demonstrating moderate discrimination, good calibration, and robust performance across diverse patient subgroups. The clinical utility of the risk score, evidenced by decision curve analysis and made accessible via an interactive web-based calculator, supports its potential for integration into clinical practice to guide personalized posttransplant management and facilitate shared decision-making.

## Notes

Supplement 1.
eMethods
**eFigure 1.** Kaplan-Meier Estimates of Event-Free Survival for the Entire Cohort **eFigure 2.** Parsimony Plot Demonstrating Model Performance vs Complexity **eFigure 3.** Calibration Plots for the Prognostic Model in the Training and Testing Cohorts **eFigure 4.** Calibration Plots of the Prognostic Model in the Testing Data Across Age Groups **eFigure 5.** Calibration-in-the-Large Analysis (Expressed as Observed/Expected Ratio) by Age Groups **eFigure 6.** Calibration Plots of the Prognostic Model in the Testing Data Across OPTN Regions **eFigure 7.** Calibration-in-the-Large Analysis (Expressed as Observed/Expected Ratio) by Transplant-Regions **eFigure 8.** Interactive User-Friendly Web-Based Risk Calculator Interface and Model Outputs **eTable 1. **Definitions of Variables Used in the Analysis **eTable 2. **Baseline Characteristics of Patients in the Study Cohorts **eTable 3. **Clinical Outcomes and Follow-Up Duration by Cohorts **eTable 4. **Univariable and Multivariable Cox Regression Analysis **eTable 5. **Time-Dependent Model Discrimination by Transplant Regions **eTable 6. **Overall Model Discrimination Performance by Transplant Regions **eTable 7. **Time-Dependent Model Discrimination by Age Groups **eTable 8. **Overall Model Discrimination Performance by Age Groups **eTable 9. **Comparison of Risk Prediction Models for Lung Transplant Outcomes
eReferences

Supplement 2.
Data Sharing Statement
