# Supplementary material for: Development and Validation of a Hybrid Machine Learning Model to Predict Lung Transplant Outcomes
Source: JAMA Netw Open. 2025 Nov 25;8(11):e2545369. doi: 10.1001/jamanetworkopen.2025.45369 (PMC12648352; doi:10.1001/jamanetworkopen.2025.45369)
Supplement: Supplement 1. — eMethods eFigure 1. Kaplan-Meier Estimates of Event-Free Survival for the Entire Cohort eFigure 2. Parsimony Plot Demonstrating Model Performance vs Complexity eFigure 3. Calibration Plots for the Prognostic Model in the Training and Testing Cohorts eFigure 4. Calibration Plots of the Prognostic Model in the Testing Data Across Age Groups eFigure 5. Calibration-in-the-Large Analysis (Expressed as Observed/Expected Ratio) by Age Groups eFigure 6. Calibration Plots of the Prognostic Model in the Testing Data Across OPTN Regions eFigure 7. Calibration-in-the-Large Analysis (Expressed as Observed/Expected Ratio) by Transplant-Regions eFigure 8. Interactive User-Friendly Web-Based Risk Calculator Interface and Model Outputs eTable 1. Definitions of Variables Used in the Analysis eTable 2. Baseline Characteristics of Patients in the Study Cohorts eTable 3. Clinical Outcomes and Follow-Up Duration by Cohorts eTable 4. Univariable and Multivariable Cox Regression Analysis eTable 5. Time-Dependent Model Discrimination by Transplant Regions eTable 6. Overall Model Discrimination Performance by Transplant Regions eTable 7. Time-Dependent Model Discrimination by Age Groups eTable 8. Overall Model Discrimination Performance by Age Groups eTable 9. Comparison of Risk Prediction Models for Lung Transplant Outcomes eReferences [file jamanetwopen-e2545369-s001.pdf]

Supplementary Online Content

Sharma G, Kamal VK, Bollineni S, et al. Development and validation of a hybrid machine learning model to predict lung transplant outcomes. *JAMA Netw Open.* 2025;8(11):e2545369. doi:10.1001/jamanetworkopen.2025.45369

eMethods
eFigure 1. Kaplan-Meier Estimates of Event-Free Survival for the Entire Cohort
eFigure 2. Parsimony Plot Demonstrating Model Performance vs Complexity
eFigure 3. Calibration Plots for the Prognostic Model in the Training and Testing Cohorts
eFigure 4. Calibration Plots of the Prognostic Model in the Testing Data Across Age Groups
eFigure 5. Calibration-in-the-Large Analysis (Expressed as Observed/Expected Ratio) By Age Groups
eFigure 6. Calibration Plots of the Prognostic Model in the Testing Data Across OPTN Regions
eFigure 7. Calibration-in-the-Large Analysis (Expressed as Observed/Expected Ratio) by Transplant-Regions
eFigure 8. Interactive User-Friendly Web-Based Risk Calculator Interface and Model Outputs
eTable 1. Definitions of Variables Used in the Analysis
eTable 2. Baseline Characteristics of Patients in the Study Cohorts
eTable 3. Clinical Outcomes and Follow-Up Duration by Cohorts
eTable 4. Univariable and Multivariable Cox Regression Analysis
eTable 5. Time-Dependent Model Discrimination by Transplant Regions
eTable 6. Overall Model Discrimination Performance by Transplant Regions
eTable 7. Time-Dependent Model Discrimination by Age Groups
eTable 8. Overall Model Discrimination Performance by Age Groups
eTable 9. Comparison of Risk Prediction Models for Lung Transplant Outcomes
eReferences

This supplementary material has been provided by the authors to give readers additional information about their work.

eMethods

S1. Sample size calculation for model development

A large sample size is essential for prognostic modeling because it improves reliability and generalizability, reduces prediction uncertainty, and limits overfitting. With enough data, especially enough events, model coefficients are more stable, risk estimates are better calibrated, and performance metrics are more trustworthy. Conversely, small samples produce unstable, inaccurate predictions, widen confidence intervals, and make it hard to assess or improve fairness and performance across key subgroups. Currently, there is no established sample size formula for hybrid machine learning models for survival outcomes. However, for traditional survival analysis-based prediction models, a sample size calculation method has been proposed by Riley et al.[1] We estimated the minimum required sample size for developing a survival prediction model for a five-year time-to-event outcome using the ‘pmsampsize’ package in R, following Riley et al.'s methodology. This approach accounts for context-specific factors, including the number of predictor parameters, event rate, follow-up duration, and anticipated model performance.

The calculation was performed using the following assumptions:

a) 24 predictor parameters (Using variables from the previous parsimonious model)
b) Event rate = 0.57 (57%) (Derived from UNOS data)
c) 5-year prediction time-point (Time to the event of interest)
d) Mean follow-up time = 4.7 years (Estimated based on previous studies)

© 2025 Sharma G et al. *JAMA Network Open.*

e) Cox-Snell R^2 (CS R-squared) = 0.0075, corresponding to Nagelkerke's R^2 (Nag_Rsq) = 0.10 (Assumed from prior experience)

Based on these inputs, the minimum required sample size was 28,680 participants, ensuring sufficient statistical power and precision for model development. This corresponds to 134,796 person-years of follow-up and 76,834 outcome events, resulting in an events-per-predictor parameter (EPP) of 3,201.41, exceeding conventional modelling standards. The sample size calculation was based on three key criteria:

a) Minimizing overfitting, ensuring predictor effect shrinkage remains within 10%.
b) Ensuring a small absolute difference (≤0.05) between the model's apparent and adjusted Nagelkerke's R^2.
c) Providing a precise estimation of the average outcome risk, with a 95% confidence interval ranging from 0.941 to 0.943, with n = 28,680.

Thus, a sample size of 28,680 participants was considered necessary to develop a robust and well-calibrated survival model, ensuring sufficient follow-up and event rates to support statistical validity and clinical applicability.

Based on our priori sample-size calculation, as mentioned above, for time-to-event prediction modelling, a minimum development sample of 28,680 was targeted to achieve adequate shrinkage and precise calibration of 1-, 5-, and 10-year risks. To enable temporal validation, we applied a chronological split of the UNOS cohort—1987–2014 for model development and 2015–2025 for testing, so that performance could be assessed on later calendar years with sufficient follow-up at each horizon. This yielded 24,014 individuals for model development (slightly below target). Within the development set, we

implemented a 90:10 random split (training:validation). A validation dataset was needed to tune model parameters and guard against overfitting. Although the development sample (n=24,014) was below the planned 28,680, design choices (temporal split, validation, constrained model complexity, and recalibration) were used to minimize any loss of precision or calibration.

S2. Handling of missing data

In the UNOS registry, missingness is explicitly coded as Not reported (NR), Unknown (U), or Not applicable (NA). For categorical predictors, we retained these codes as substantive levels to preserve the registry's semantics and avoid listwise deletion; this was done for all categorical variables irrespective of the missing proportion. When any of NR/U/NA categories were very sparse, we consolidated them into a single level in combination with any two or all three of the "NR/U/NA" level; if still sparse, we merged the level with the most clinically appropriate category to prevent small-cell instability. For continuous predictors, if the proportion missing was ≤5%, we applied sex-stratified median imputation; if >5%, we preserved missingness by creating an explicit NR indicator for that variable and including this indicator in the model (in addition to the observed values), thereby maintaining the original UNOS coding while limiting bias from case-wise deletion. All missing data decisions were prespecified and applied uniformly across candidate predictors.

S3. Statistical analysis

Descriptive statistics for baseline characteristics were presented as a median with 5th and 95th percentiles as the quartile range for continuous variables and frequencies with

percentages for categorical variables. Univariable and multivariable Cox regression analyses were used to assess associations between candidate variables and the primary outcome. Associations were quantified using unadjusted and adjusted hazard ratios (95% CI). Kaplan-Meier survival curves for the entire cohort were also constructed to illustrate survival differences across risk groups, and the log-rank test was applied for statistical comparison. All statistical analyses were performed using R software (version 4.4.1; R Foundation for Statistical Computing, Vienna, Austria), except for calibration plots and decision curve analysis, which were generated using Stata software (version 17.0; StataCorp LLC, College Station, TX, USA). A two-sided significance level was set at $P < 0.05$.

S4. Model development

The AutoScore-Survival methodology[2,3] comprises six distinct modules, which were sequentially applied to the training dataset. In the first module, a random survival forest algorithm was used to rank candidate variables according to their predictive importance. Variables that contributed minimally to the survival outcome were eliminated from subsequent analyses. In the second module, continuous variables were discretized into clinically meaningful categories using data-driven thresholds, ensuring that the resulting categories reflected real-world clinical decision points. In the third module, a Cox proportional hazards regression was applied to the training data to assign weights to the selected variables. The regression coefficients were normalized and converted into integer scores, thereby creating a transparent scoring system that could be easily implemented in clinical practice. The fourth module involved the generation of a parsimony plot, which depicted the trade-off between model complexity and predictive

© 2025 Sharma G et al. *JAMA Network Open.*

accuracy using a validation cohort, guiding the selection of the final model. In the fifth module, cutoffs for variable discretization were fine-tuned through iterative analysis to optimize calibration. Cutoff values were determined by a combination of data-driven analysis and clinical relevance. Finally, the sixth module entailed the evaluation of the final model's performance in the testing dataset using time-dependent Area Under the Curve (AUC (t)), C-index (Harrell's Concordance Index), and integrated AUC (iAUC) metrics.

We developed an interpretable time-to-event prediction tool for lung transplant patients using UNOS registry data (n=51,933), following a structured machine learning and Cox regression-based approach:

1. Dataset Partitioning: The dataset was temporally split into development (1987-2014; n=26,682) and testing (2015-2025; n=25,251) sets. The development set was further randomly split into training (90%, n=24,014) and validation (10%, n=2,668) sets. The training set was used for model development, the validation set for parameter tuning and model selection, and the testing set, an unseen dataset, for final performance assessment.
2. Variable Selection: The training cohort underwent feature selection using a random survival forest (500 trees), where candidate variables were ranked based on their impact on predictive accuracy, determined by their permutation importance score.
3. Variable Transformation: Selected continuous variables were automatically categorized using quantile-based discretization, ensuring clinically meaningful risk stratification.

4. Score Generation: Cox regression was applied to assign weights to variable categories, ensuring non-negative scores by setting the reference category to the one with the smallest β coefficient. A second-step regression was performed to generate final coefficients, which were normalized, rounded to the nearest integer, and assigned as partial scores. The total risk score was calculated by summing all partial scores.
5. Model Parsimony and Selection: The number of variables in the final model was determined using a parsimony plot, balancing predictive accuracy (AUC(t)) and model complexity on the validation cohort.
6. Cutoff Optimization: Variable cutoffs and final scoring thresholds were fine-tuned based on validation performance to maximize discrimination.
7. Risk Probability Estimation: The final risk model was incorporated after recalibration using the 2020-2025 testing data into a web-based risk calculator, using the following regression equations for death/retransplantation probability estimation:

   $P(\text{1-Year}) = 1 - 0.9542952^{\exp(lp)}$

   $P(\text{5-Years}) = 1 - 0.7946746^{\exp(lp)}$

   $P(\text{10-Years}) = 1 - 0.4926291^{\exp(lp)}$

   Where: $lp = 0.0278129 \times \text{total score}$

8. Model Performance Evaluation: The discrimination (AUC, Harrell's C-index) and calibration of the model were assessed in both training and testing datasets.
9. Clinical Utility Assessment: Decision curve analysis was conducted on the testing dataset to evaluate the net benefit of the scoring system in clinical decision-making.

© 2025 Sharma G et al. *JAMA Network Open.*

This structured approach ensured the development of a parsimonious, interpretable, and clinically applicable risk prediction model for lung transplant recipients.

S5. Purposes of training, validation, and testing cohort

The training cohort was used to develop the model by identifying patterns and relationships between predictor variables and the outcome. Within the AutoScore framework, this dataset facilitated variable selection, score assignment, and preliminary model construction. The validation cohort was utilized to fine-tune the model, optimize hyperparameters, refine score cutoffs, and select the most parsimonious model before final testing. This step was essential in preventing overfitting, as it allowed assessment of data that was not directly used for model training.

The testing cohort, an independent unseen dataset, was used for final evaluation to assess the model's generalizability and real-world performance. This step ensured that the model maintained consistent predictive accuracy on new data and was not overfitted to the training dataset.

S6. Temporal Recalibration and Model Updating

To account for calendar-time shifts in baseline risk, we performed recalibration-in-the-large on the temporal validation cohort (2015–2025).[4] For each individual, we computed the original linear predictor from the development model ($lp = X\hat{\beta}$, trained on 1987–2014) and then fit a Cox model with lp entered as an offset (coefficient fixed to 1) on the temporal data to re-estimate the baseline cumulative hazard $H0^*(t)$. Recalibrated survival and risk were obtained as $S^*(t \mid X) = \exp\{-H0^*(t) \times \exp(lp)\}$ and $P^*(t \mid X) = 1 - S^*(t \mid X)$, thereby keeping $\hat{\beta}$ unchanged and updating only the baseline.

© 2025 Sharma G et al. *JAMA Network Open.*

We again reassessed model performance after recalibration, discrimination (Harrell's C and time-dependent AUC at 1, 5, and 10 years) and calibration (calibration-in-the-large observed/expected ratios, calibration plots, and Brier scores), overall and in prespecified subgroups by UNOS transplant region and by age bands (<40, 40–60, >60) to evaluate robustness.

S7. Clinical Utility (Decision Curve Analysis)

We used Decision Curve Analysis (DCA) to show whether using the model to guide decisions would help patients more than simply treating everyone or treating no one. DCA plots net benefit across a range of risk thresholds, the cut-offs at which a clinician would choose to act (e.g., if predicted 5-year risk ≥ 30%). A higher curve indicates greater clinical value after balancing true positives against the harm of unnecessary actions in people who would not experience the outcome. If the model's curve lies above both the "treat all" and "treat none" lines over thresholds that match real-world practice, then using the model would improve decision-making. For context, the vertical axis can be interpreted as the number of unnecessary interventions avoided per 100 patients (after accounting for missed cases). We present DCA at the relevant time horizons (1, 5, and 10 years) to illustrate where the model is most useful in practice.

S8. Methodological Decisions for Model Robustness and Clinical Relevance

Methodological Revisions in Response to Peer Review: In the initial analysis, a random data split was used for model development and validation. However, based on critical reviewer feedback highlighting the importance of assessing model performance over time, we performed a complete re-analysis. The revised manuscript now employs a

© 2025 Sharma G et al. *JAMA Network Open.*

strict temporal validation strategy, with the dataset partitioned into a development cohort (transplants 1987–2014) and a temporal testing cohort (2015–2025). This approach provides a more rigorous and clinically meaningful evaluation of the model's generalizability and robustness to secular trends in patient characteristics and clinical practice, directly addressing a key limitation of the original submission and significantly strengthening the study's conclusions.

Variable Selection and Stability: To ensure the stability of the variable selection process, the random survival forest algorithm (500 trees) was run five times on the training cohort. The top 10 predictors were consistently identified across all iterations, with only minor variations in their relative importance ranking. For instance, donor age consistently ranked between 8th and 10th. Including variables beyond this top set offered only marginal gains in the integrated Area Under the Curve (iAUC). Therefore, a more parsimonious nine-variable model was selected to optimize clinical usability without sacrificing meaningful predictive power. The model's performance remained robust across the training and temporal testing cohorts after recalibration, indicating its stability against temporal drift in the data.

Modeling Strategy for Clinical Realism: A fundamental decision in this study was to model the risk of death or re-transplantation from the moment of transplant (day zero), rather than from the time of hospital discharge. This approach was chosen to avoid two key methodological challenges:

Survivorship Bias: Modeling only patients who survive to discharge would introduce a severe survivorship bias. A model trained exclusively on this cohort of "successful" initial outcomes would be dangerously optimistic and clinically misleading, as it would

systematically underestimate risk by ignoring the group with the highest early hazard. For a prognostic tool to be clinically responsible and useful for shared decision-making, it must reflect the complete risk profile a patient faces.

Incorporation Bias: While essential for clinical honesty, the "day zero" approach necessitates careful management of incorporation bias, where an early outcome (e.g., in-hospital death) can improperly influence a predictor (e.g., Length of Stay). To mitigate this, we identified and excluded 170 index-hospitalization deaths or re-transplants from the analysis. However, this exclusion was incomplete due to missing discharge dates in some records. This residual misclassification likely explains the strong predictive power of a very short length of stay (<6 days), which serves as a proxy for unmeasured, catastrophic early events.

Model Calibration in Specific Subgroups

The model demonstrated robust calibration at 1 and 5 years across the full testing cohort. However, in subgroup analyses, particularly at the 10-year horizon, calibration plots showed increased variability. This was most evident in strata with fewer events and heavier censoring, such as the <40 and >60 age bands and certain OPTN regions, resulting in wider confidence intervals and less stable Kaplan-Meier estimates. While we recalibrated the model's baseline hazard on the temporal cohort, covariate effects were not re-estimated, which may contribute to residual miscalibration in specific subgroups over longer follow-up periods.

Methodological Rigor and Reporting: The methodological framework for this study was designed for rigor and transparency. The entire analysis, from development to reporting,

© 2025 Sharma G et al. *JAMA Network Open.*

adheres to the TRIPOD-AI statement. The use of a strict temporal validation split, comprehensive assessments of both discrimination and calibration at multiple time horizons, and the evaluation of clinical utility via Decision Curve Analysis provide a robust, end-to-end evaluation of the model's performance and potential for real-world clinical adoption.

© 2025 Sharma G et al. *JAMA Network Open.*

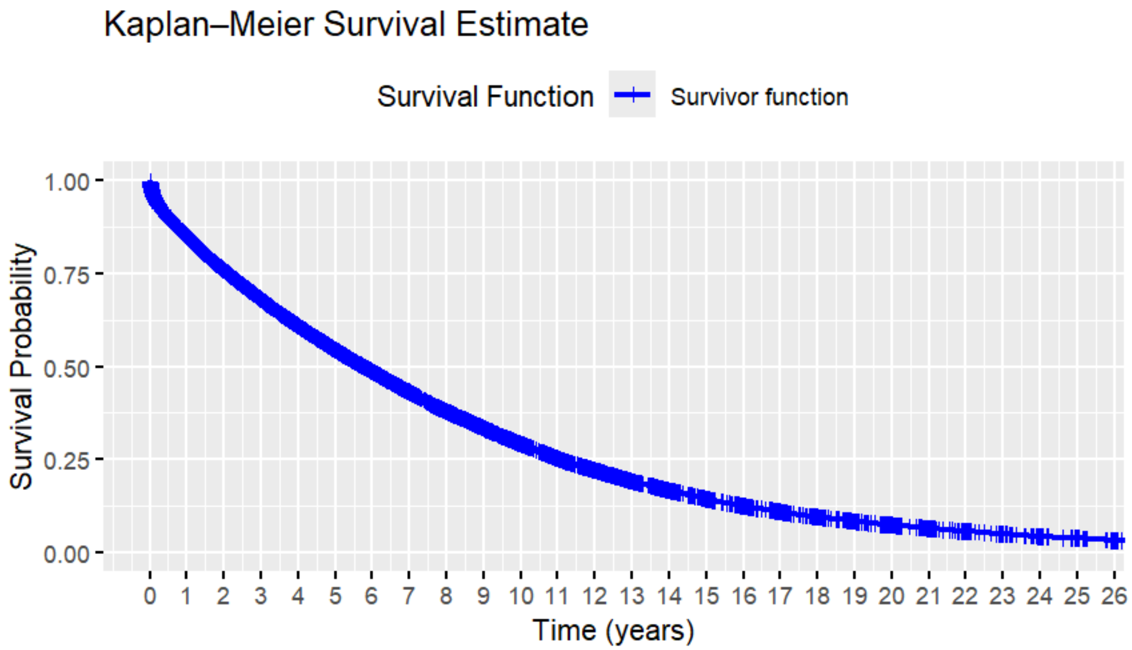

eFigure 1. Kaplan-Meier Estimates of Event-Free Survival for the Entire Cohort Kaplan-Meier survival curves depicting the cumulative probability of remaining free from death or re-transplantation over time in the full cohort of lung transplant recipients (N=51,933). Shaded areas represent 95% confidence intervals.

© 2025 Sharma G et al. *JAMA Network Open.*

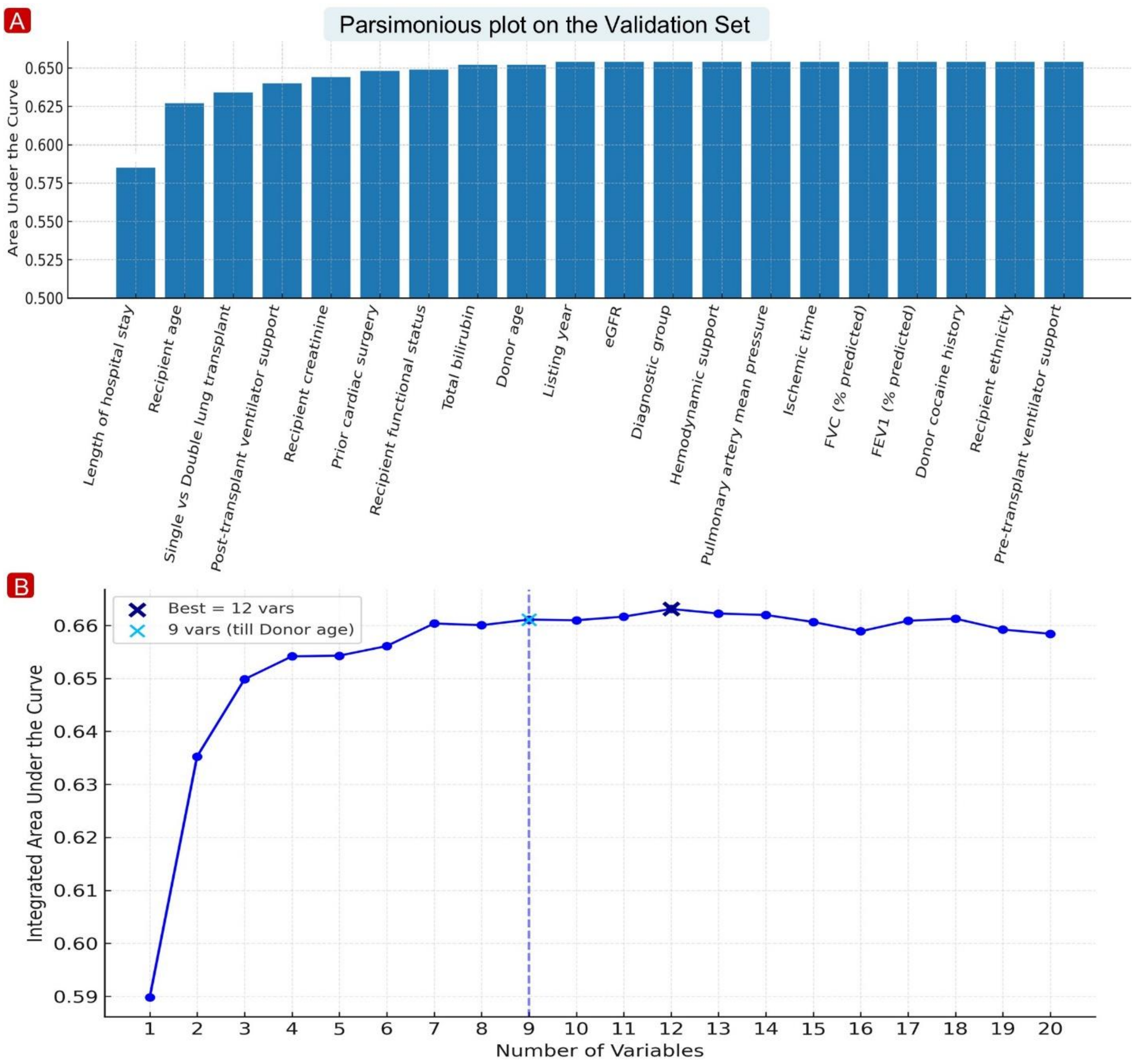

eFigure 2. Parsimony Plot Demonstrating Model Performance vs Complexity

A plot illustrating the trade-off between model complexity (number of included variables) and predictive performance, as measured by the integrated area under the curve (iAUC) in the validation cohort. Each point represents a model iteration with a specific set of variables, guiding the selection of an optimal, parsimonious model.

© 2025 Sharma G et al. *JAMA Network Open.*

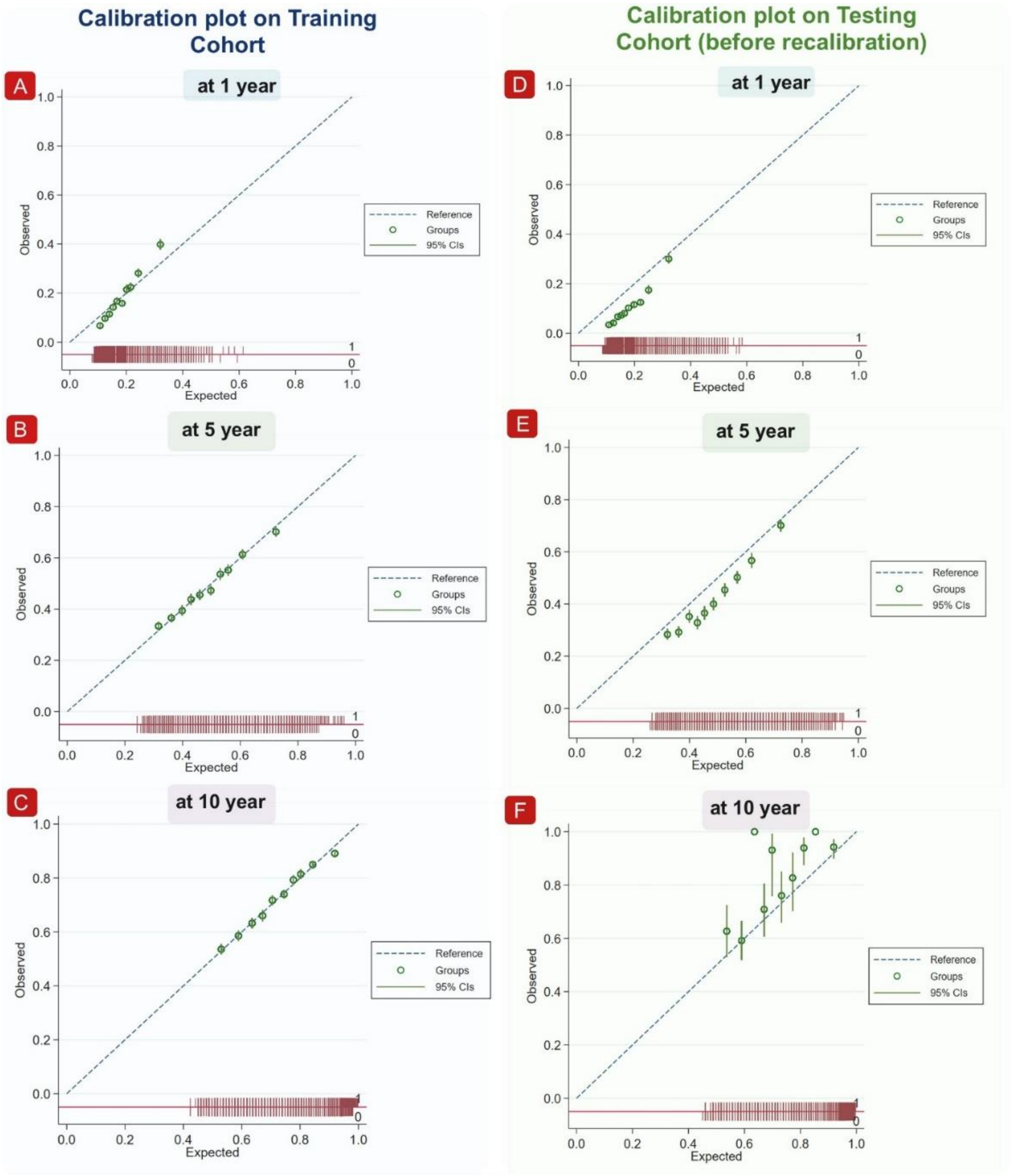

eFigure 3. Calibration Plots for the Prognostic Model in the Training and Testing Cohorts

Calibration plots demonstrate the agreement between predicted and observed risk probabilities in the training cohort (A-C) and the testing cohort before recalibration (D-F) at 1 year (A, D), 5 years (B, E), and 10 years (C, F). The dashed line represents perfect calibration. Green circles indicate deciles of predicted risk, with error bars showing 95% confidence intervals. The rug plots at the bottom display the distribution of predicted probabilities.

© 2025 Sharma G et al. *JAMA Network Open.*

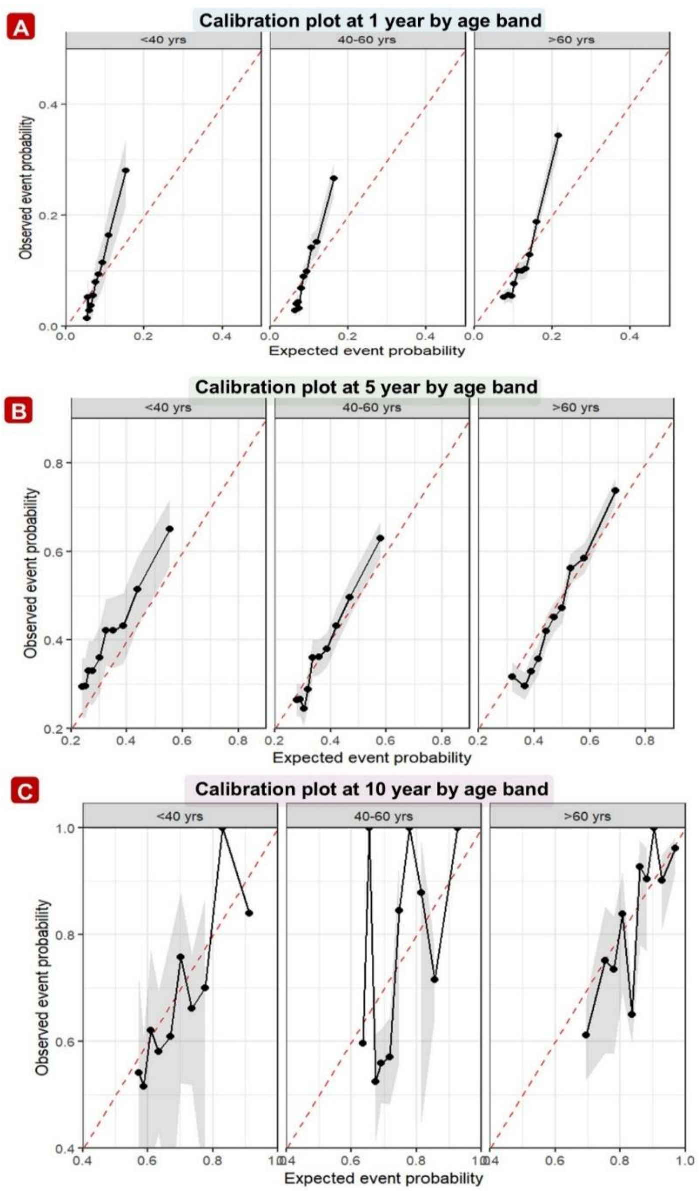

eFigure 4. Calibration Plots of the Prognostic Model in the Testing Data Across Age Groups

Calibration Plots at (A) 1 Year, (B) 5 Years, and (C) 10 Years, Stratified by Age Categories (<40 Years, 40–60 Years, and >60 Years). The red dashed line represents perfect calibration, assessing the model's performance.

© 2025 Sharma G et al. *JAMA Network Open.*

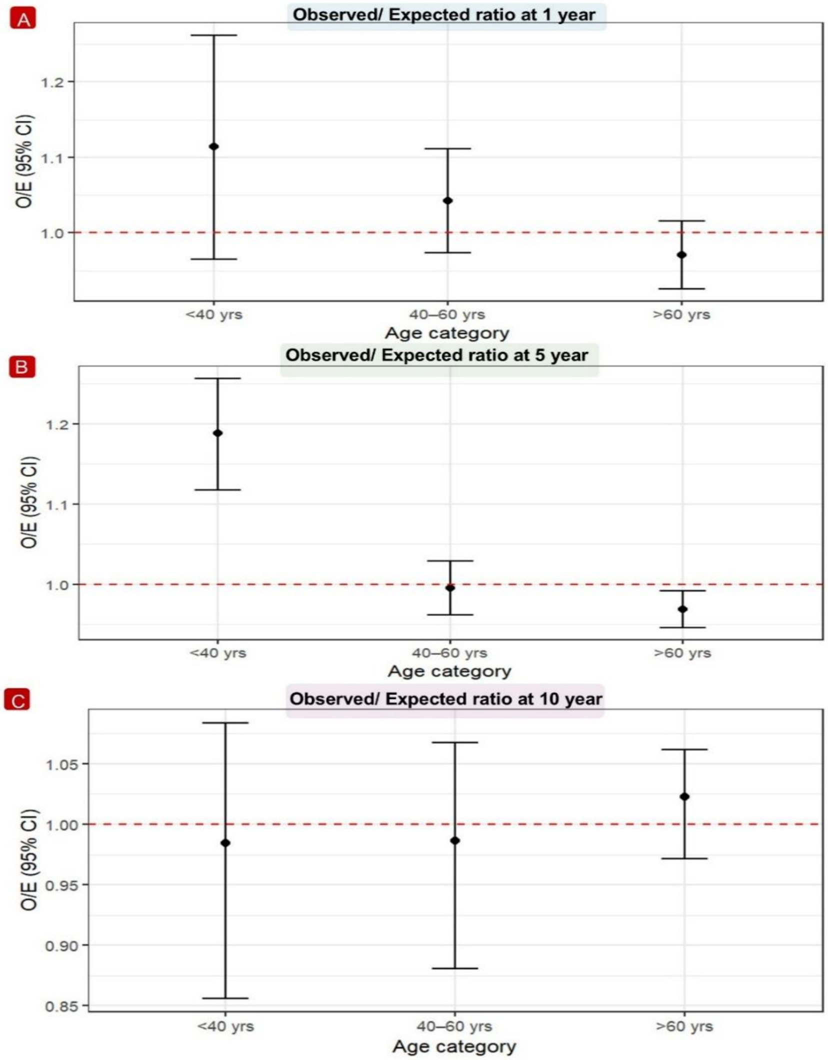

eFigure 5. Calibration-in-the-Large Analysis (Expressed as Observed/Expected Ratio) by Age Groups

Observed-to-Expected (O/E) ratios with 95% confidence intervals (CIs) for death or re-transplantation at (A) 1-year, (B) 5-year, and (C) 10-year horizons, stratified by age categories (<40 years, 40–60 years, and >60 years). The red dashed line at O/E = 1.0 indicates perfect calibration-in-the-large.

© 2025 Sharma G et al. *JAMA Network Open.*

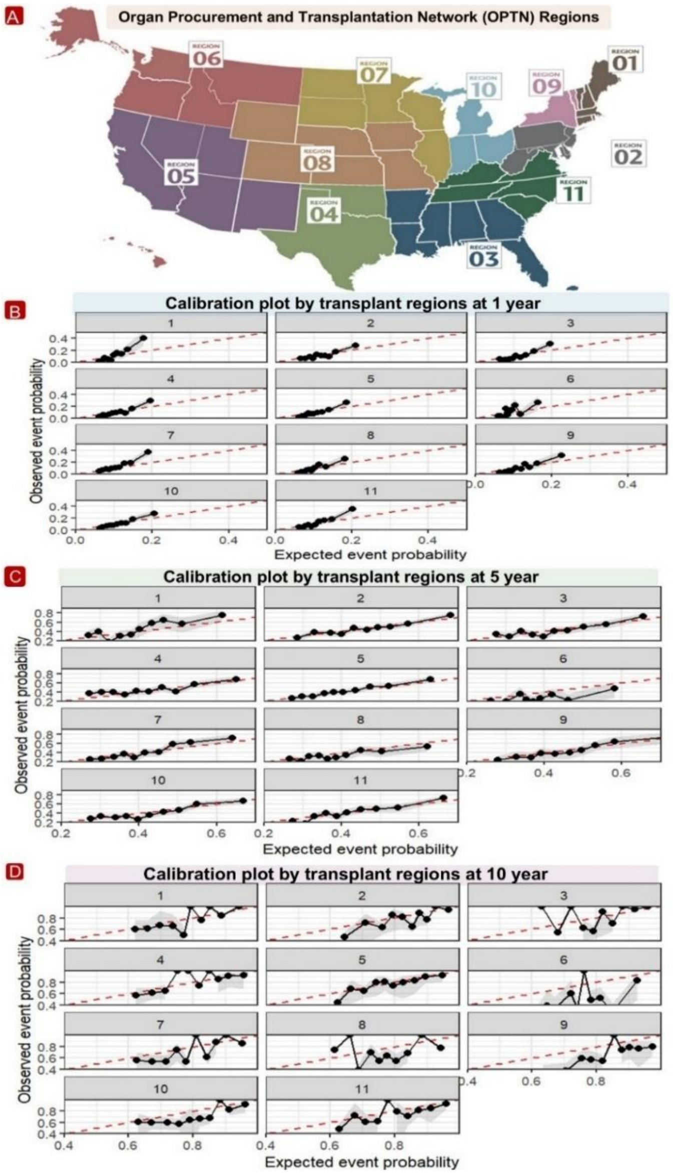

eFigure 6. Calibration Plots of the Prognostic Model in the Testing Data Across OPTN Regions

(A) Map of the 11 Organ Procurement and Transplantation Network (OPTN) regions in the United States. (B-D) Calibration plots for the Prognostic model stratified by each OPTN region at 1 year (B), 5 years (C), and 10 years (D). The red dashed line represents perfect calibration, assessing the model's performance across different transplant regions.

© 2025 Sharma G et al. *JAMA Network Open.*

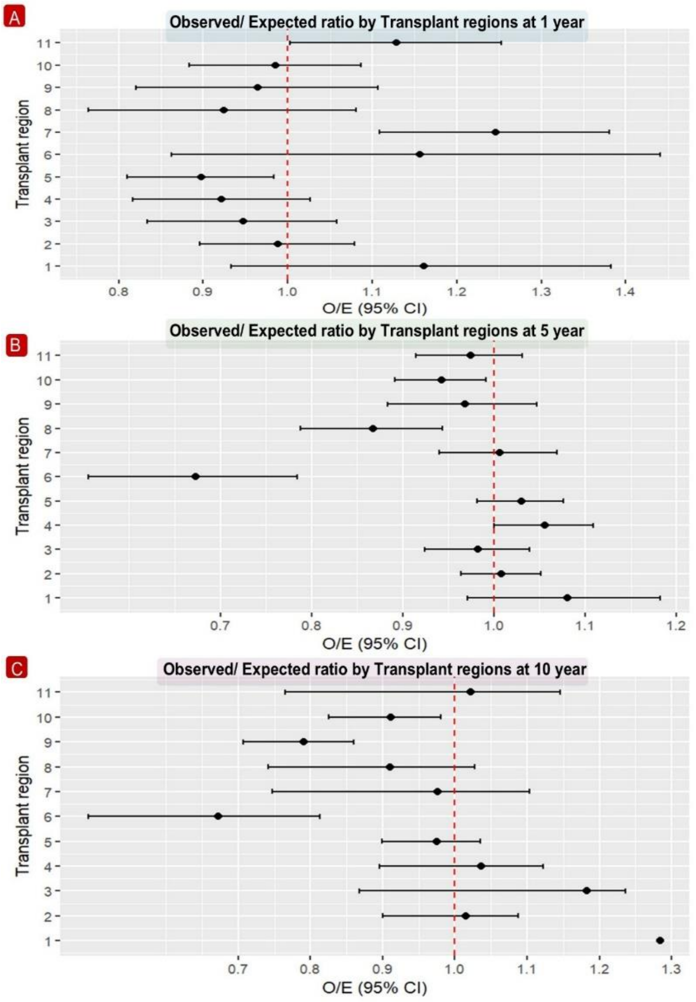

eFigure 7. Calibration-in-the-Large Analysis (Expressed as Observed/Expected Ratio) by Transplant-Regions

Observed-to-Expected (O/E) ratios with 95% confidence intervals (CIs) for death or re-transplantation at (A) 1-year, (B) 5-year, and (C) 10-year horizons, stratified by the 11 Organ Procurement and Transplantation Network (OPTN) regions. The red dashed line at O/E = 1.0 indicates perfect calibration-in-the-large.

© 2025 Sharma G et al. *JAMA Network Open.*

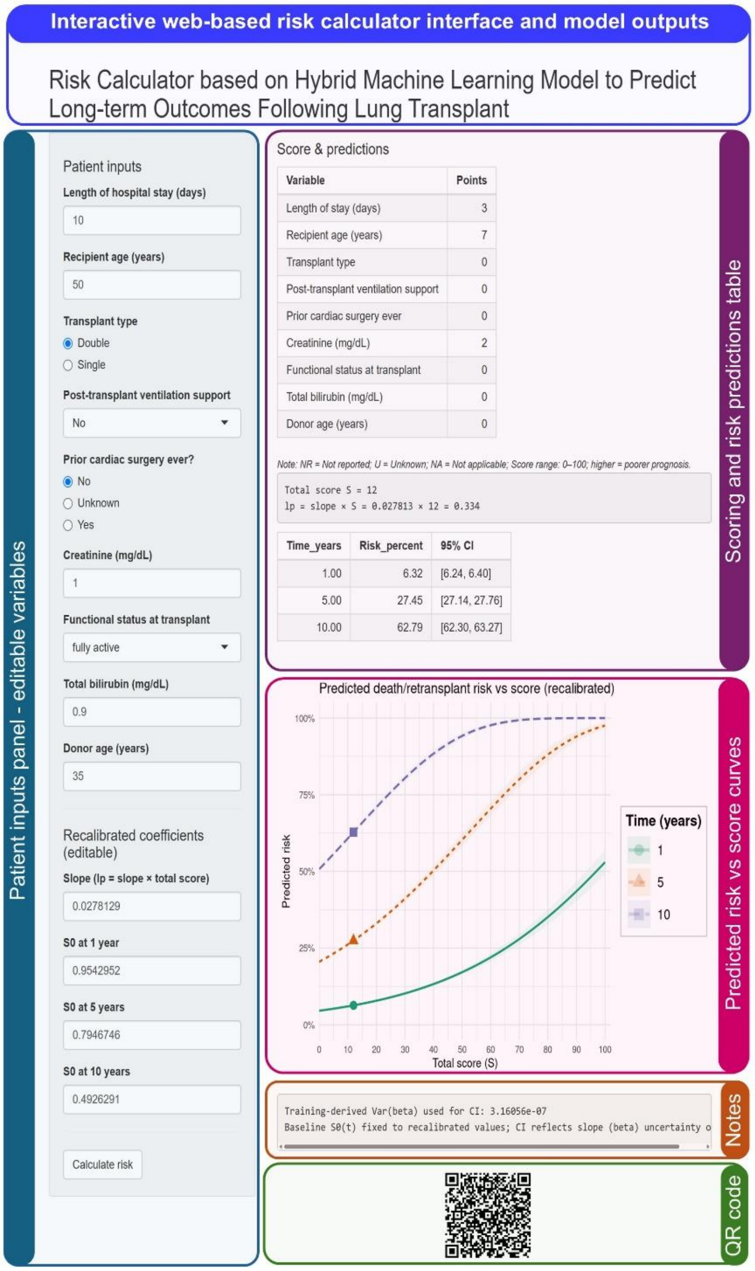

eFigure 8. Interactive User-Friendly Web-Based Risk Calculator Interface and Model Outputs

Left panel shows user-editable patient inputs and recalibrated coefficients (slope and baseline survival S0 at 1, 5, 10 years). Center top displays the scoring table with per-variable points and the computed total score S. The linear predictor lp = slope×S is

© 2025 Sharma G et al. *JAMA Network Open.*

calculated and combined with recalibrated baseline survival to produce predicted probabilities for death or retransplant. Center table reports risk (%) and 95% confidence intervals at 1, 5, and 10 years for the example patient. Lower right shows predicted risk versus total score with separate curves for 1, 5, and 10 years; filled markers indicate the current patient score. Bottom text lists technical calibration parameters and variances used for CI estimation. QR code links to the online calculator. Available at https://risk-calculator12.shinyapps.io/lung-transplant-risk/

© 2025 Sharma G et al. *JAMA Network Open.*

eTable 1: Definitions of variables used in the analysis

S. No.	Variable Name (in our data set)\	Variable Name (in UNOS data)	Description (in UNOS data)	General Description	Label
**Recipient-Level**					
1	age_rec	AGE	RECIPIENT AGE (YRS)	Recipient Age	Age
2	gen_rec	GENDER	RECIPIENT GENDER	Recipient Gender	Gender
3	eth_rec	ETHCAT	RECIPIENT ETHNICITY CATEGORY	Recipient Ethnicity	Ethnicity
4	bmi_rec	BMI_CALC	Calculated Recipient BMI	Recipient Body Mass Index	BMI
5	int_las_rec_clean	INIT_CALC_LAS	INITIAL CALCULATED LUNG ALLOCATION SCORE	Initial Lung Allocation Score Category for Recipient	Initial LAS Category
6	end_las_rec_clean	END_CALC_LAS	REMOVAL/CURRENT CALCULATED LUNG ALLOCATION SCORE	Final Lung Allocation Score Category for Recipient	Final LAS Category
7	init_match_las_clean	INIT_MATCH_LAS	INITIAL LUNG ALLOCATION SCORE AT MATCH TIME	Initial Matched Lung Allocation Score Category	Initial Matched LAS Category
8	end_match_las_clean	END_MATCH_LAS	REMOVAL/CURRENT LUNG ALLOCATION SCORE AT MATCH TIME	Final Matched Lung Allocation Score Category	Final Matched LAS Category
9	fun_stat_regt_rec_clean	FUNC_STAT_TCR	RECIPIENT FUNCTIONAL STATUS @ REGISTRATION	Functional Status at Registration for Recipient	Functional Status at Registration
10	fun_stat_tx_rec_clean	FUNC_STAT_TRR	RECIPIENT FUNCTIONAL STATUS @TRANSPLANT	Functional Status at Transplant for Recipient	Functional Status at Transplant
11	chro_stroid_use_rec_clean	STEROID	CHRONIC STEROID USE Y/N/U @ TRANSPLANT	Chronic Steroid Use for Recipient	Chronic Steroid Use
12	trr_lifesupport_vent_clean	VENTILATOR_TRR	PATIENT ON LIFE SUPPORT - VENTILATOR @ TRANSPLANT	Life Support Ventilation at Transplant	Life Support Ventilation
13	cmv2_rec_clean	CMV_STATUS	RECIPIENT CMV Status @ TRANSPLANT	CMV Status for Recipient	CMV Status
14	payment_source_rec_clean	PRI_PAYMENT_TRR	RECIPIENT PRIMARY PAYMENT SOURCE @ TRANSPLANT	Payment Source for Transplant	Payment Source
15	hlamismatch_cat	HLAMIS	HLA MISMATCH LEVEL	HLA Mismatch Category	HLA Mismatch Category
16	diab_rec_clean	DIAB	RECIPIENT DIABETES @ REGISTRATION	Diabetes Status for Recipient	Diabetes

© 2025 Sharma G et al. *JAMA Network Open.*

eTable 1: Definitions of variables used in the analysis (contd..)

S. No.	Variable Name (in our data set)\	Variable Name (in UNOS data)	Description (in UNOS data)	General Description	Label
**Recipient-Level**					
17	grouping_clean	GROUPING	LU/HL DIAGNOSIS GROUPING ON WL(ON THORACIC_DGN/TCR_TGN/TRR_DGN)	Grouping Category	Grouping Category
18	pan_bactr_lung_inf_rec	RESIST_INF	PAN-RESISTANT BACTERIAL INFECTION @ REGISTRATION	Pan-Bacterial Lung Infection for Recipient	Pan-Bacterial Lung Infection
19	drugtherapy_2weekprior_rec	INFECT_IV_DRUG_TRR	INFECTION REQUIRING IV DRUG THERAPY (WITHIN 2 WEEKS PRIOR TO TRANSPLANT)	Drug Therapy within 2 Weeks Prior to Transplant	Drug Therapy 2 Weeks Prior
20	cig_use_rec_clean	CIG_USE	HISTORY OF CIGARETTE USE	Cigarette Use for Recipient	Cigarette Use
21	fev_rec	FEV1_TRR	PULMONARY STATUS: FEV1 % PREDICTED @ TRANSPLANT	Forced Expiratory Volume for Recipient	FEV
22	acute_rej_episode_clean	ACUTE_REJ_EPI	DID RECIPIENT HAVE ANY ACUTE REJECTION EPISODES PRE DISCHARGE?	Acute Rejection Episodes for Recipient	Acute Rejection Episodes
23	multoorgan_transplant_clean	MULTIORG	MULTI-ORGAN TRANSPLANT	Multi-Organ Transplant Status	Multi-Organ Transplant
24	inotrop_meanarterypres_rec_clean	INOTROP_VASO_MN_TRR	TRR MEAN PULMONARY ARTERY MEASUREMENT OBTAINED WHILE ON INOTROPES OR VASODILATERS Y/N	Inotropic Support with Mean Arterial Pressure for Recipient	Inotropic Support with MAP
25	fvc_rec	FVC_TRR	PULMONARY STATUS: FVC % PREDICTED @ TRANSPLANT	Forced Vital Capacity for Recipient	FVC
26	pre_tx_dia_ever_clean	Derived using (1. DIAL_AFTER_LIST, 2. DIAL_PRIOR_TX)	DIALYSIS OCCURRING BETWEEN LISTING AND TRANSPLANT, Calculated: Ever Dialysis Prior Tx?	Pre-Transplant Dialysis Status Ever (Indicates whether the recipient received dialysis at any time before the lung transplant, covering both (a) dialysis prior to transplant and (b) dialysis after listing up to the time of transplant.	Pre-Transplant Dialysis

© 2025 Sharma G et al. *JAMA Network Open.*

eTable 1: Definitions of variables used in the analysis (contd..)

S. No.	Variable Name (in our data set)\	Variable Name (in UNOS data)	Description (in UNOS data)	General Description	Label
**Recipient-Level**					
27	prior_car_sur_ever	Derived using (1. PRIOR_CARD_SURG_TRR, 2. PRIOR_CARD_SURG_TCR)	TRR CARDIAC SURGERY BETWEEN LISTING AND TRANSPLANT (NON-TRANSPLANT), TCR PRIOR CARDIAC SURGERY AT LISTING (NON-TRANSPLANT)	Indicates whether the recipient had any cardiac surgery before the lung transplant (Prior Cardiac Surgery Status Ever)	Prior Cardiac Surgery
28	multiple_listing_withkidney	WLKI	WAS CANDIDATE LISTED FOR SIMULTANEOUS KIDNEY?	Multiple Listing with Kidney Transplant	Multiple Listing with Kidney
29	simultan_kidney_tx	TXKID	SIMULTANEOUS KIDNEY	Simultaneous Kidney Transplant	Simultaneous Kidney Transplant
30	lifesupport_trr_rec_clean	LIFE_SUP_TRR	RECIPIENT LIFE SUPPORT PRE-TRANSPLANT @ TRANSPLANT	Life Support at Transplant	Life Support at Transplant
31	episode_vent_support_clean	VENT_SUPPORT_AFTER_LIST	EVENTS OCCURRING BETWEEN LISTING AND TRANSPLANT: EPISODE OF VENTILATORY SUPPORT	Episodes of Ventilatory Support since Listing	Ventilator Support since Listing
32	listyear	LISTYR	ACTUAL YEAR REGISTRANT LISTED (WITHOUT DATE OFFSET)	Listing Year	Listing Year
33	totaldays_waitinglist	DAYSWAIT_CHRON	TOTAL DAYS ON WAITING LIST	Total Days on Waiting List	Total Days on Waiting List
34	HCV_sero_clean	HCV_SEROSTATUS	RECIPIENT HEP C STATUS	HCV Serostatus for Recipient	HCV Serostatus
35	tot_bilirubin	TBILI	MOST RECENT SERUM TOTAL BILIRUBIN @ TRANSPLANT	Serum Botal Bilirubin at transplant	Total Bilirubin
36	diag5	DIAG	RECIPIENT PRIMARY DIAGNOSIS	Primary Diagnosis Category	Diagnosis
37	creatine_trr_rec_clean	CREAT_TRR	RECIPIENT SERUM CREATININE AT TIME OF TX	Recipient Creatinine	Creatinine

© 2025 Sharma G et al. *JAMA Network Open.*

eTable 1: Definitions of variables used in the analysis (contd..)

S. No.	Variable Name (in our data set)\	Variable Name (in UNOS data)	Description (in UNOS data)	General Description	Label
**Recipient-Level**					
38	egfr_modified	Derived using (1. AGE, 2. GENDER , 3. CREAT_TRR)	Not given directly in UNOS data; Estimated glomerular filtration rate (eGFR, mL/min/1.73 m²) using the CKD-EPI 2009 creatinine-only equation (race term removed), with the female factor (×1.018) absorbed into the constant for women.	Estimated Glomerular Filtration Rate: eGFR (CKD-EPI 2009, Creatinine-only, Race-free) Notation: Scr = serum creatinine (mg/dL); Age = years; gen_rec = 1 (female), 2 (male). These formulas return eGFR in mL/min/1.73 m². Females (gen_rec = 1) eGFR = 144 × min(Scr/0.7, 1)^(−0.329) × max(Scr/0.7, 1)^(−1.209) × 0.993^Age Equivalent piecewise form: • if Scr ≤ 0.7: eGFR = 144 × (Scr/0.7)^(−0.329) × 0.993^Age • if Scr > 0.7: eGFR = 144 × (Scr/0.7)^(−1.209) × 0.993^Age Males (gen_rec = 2) eGFR = 141 × min(Scr/0.9, 1)^(−0.411) × max(Scr/0.9, 1)^(−1.209) × 0.993^Age Equivalent piecewise form: • if Scr ≤ 0.9: eGFR = 141 × (Scr/0.9)^(−0.411) × 0.993^Age • if Scr > 0.9: eGFR = 141 × (Scr/0.9)^(−1.209) × 0.993^Age	eGFR
39	ECMO_transplant_clean	ECMO_TRR	PATIENT ON LIFE SUPPORT - ECMO @ TRANSPLANT	Life Support ECMO at Transplant	ECMO at Transplant
**Donor-Level**					
40	hist_cocaine_don_clean	HIST_COCAINE_DON	DECEASED DONOR-HISTORY OF COCAINE USE IN PAST	History of Cocaine Use for Donor	Donor Cocaine History
41	age_don	AGE_DON	DONOR AGE (YRS)	Donor Age	Donor Age
42	gen_don	GENDER_DON	DONOR GENDER	Donor Gender	Donor Gender
43	bmi_don	BMI_DON_CALC	Donor BMI - Pre/At Donation Calculated	Donor Body Mass Index	Donor BMI

© 2025 Sharma G et al. *JAMA Network Open.*

eTable 1: Definitions of variables used in the analysis (contd..)

S. No.	Variable Name (in our data set)\	Variable Name (in UNOS data)	Description (in UNOS data)	General Description	Label
**Donor-Level**					
44	cause_death_don_clean	COD_CAD_DON	DECEASED DONOR-CAUSE OF DEATH	Cause of Death for Donor	Cause of Death
45	eth_don_clean	ETHCAT_DON	DONOR ETHNICITY CATEGORY	Donor Ethnicity	Donor Ethnicity
46	cig_use_don_clean	HIST_CIG_DON	DECEASED DONOR-HISTORY OF CIGARETTES IN PAST @ >20PACK YRS	Cigarette Use for Donor	Donor Cigarette Use
47	diab_don_clean	HIST_DIABETES_DON	DECEASED DONOR-HISTORY OF DIABETES, INCL. DURATION OF DISEASE	Diabetes Status for Donor	Donor Diabetes
48	hypertens_don_clean	HIST_HYPERTENS_DON	DECEASED DONOR-HISTORY OF HYPERTENSION	Hypertension Status for Donor	Donor Hypertension
49	alcohol_heavy_don_clean	ALCOHOL_HEAVY_DON	Heavy Alcohol Use (heavy=2+ drinks/day)	Heavy Alcohol Use for Donor	Donor Heavy Alcohol Use
50	ecd_don_clean	ECD_DONOR	EXPANDED DONOR PER KIDNEY ALLOCATION DEFINITION 1=YES	Expanded Criteria Donor Status	ECD Donor Status
**Transplant-Level**					
51	his_othdrug_don_clean	HIST_OTH_DRUG_DON	DECEASED DONOR-HISTORY OF OTHER DRUG USE IN PAST	History of Other Drug Use for Donor	Other Drug Use History
52	ischemictime_rec	ISCHTIME	ISCHEMIC TIME IN HOURS	Ischemic Time	Ischemic Time
53	singdoblung_tx_clean	TX_TYPE	TYPE OF TRANSPLANT	Single vs Double Lung Transplant	Single vs Double Lung Transplant

© 2025 Sharma G et al. *JAMA Network Open.*

eTable 1: Definitions of variables used in the analysis (contd..)

S. No.	Variable Name (in our data set)\	Variable Name (in UNOS data)	Description (in UNOS data)	General Description	Label
**Transplant-Level**					
54	hos_dis	DISTANCE	DISTANCE FROM DONOR HOSP TO TX CENTER (Nautical Miles)	Hospital Discharge Status	Hospital Discharge
55	tx_year_cat	TX_YEAR	TRANSPLANT YEAR	Transplant Year Category	Transplant Year
56	los_new_clean	Derived using (1. DISCHARGE_DATE,2. TX_DATE)	RECIPIENT DISCHARGE DATE FROM TX CENTER, TRANSPLANT DATE	Length of Hospital Stay (DISCHARGE_DATE - TX_DATE)	Length of Stay
57	posttx_ventsupp_clean	POST_TX_VENT_SUPPORT	RECIPIENT VENTILATOR SUPPORT POST TRANSPLANT	Post-Transplant Ventilator Support	Post-Transplant Ventilator Support
58	hemo_sys_trr	HEMO_SYS_TRR	MOST RECENT HEMODYNAMICS PA (SYS) MM/HG @ TRANSPLANT	Hemodynamic Support at Transplant	Hemodynamic Support
59	pa_mean_pressure	HEMO_PA_MN_TRR	MOST RECENT HEMODYNAMICS PA (MEAN) MM/HG @ TRANSPLANT	Pulmonary Artery Mean Pressure	Pulmonary Artery Mean Pressure
60	ECMO_pretransplant_rec_clean	ECMO_TRR	PATIENT ON LIFE SUPPORT - ECMO @ TRANSPLANT	ECMO at Listing	ECMO Pre-Transplant
61	transpalnt_region_clean	REGION	UNOS REGION WHERE TRANSPLANTED/ LISTED	Transplant Region	Transplant Region

© 2025 Sharma G et al. *JAMA Network Open.*

eTable 1. Definitions of Variables Used in the Analysis. This table lists the recipient-, donor-, and transplant-level variables used in the analysis with their descriptions and labels. *Abbreviations:* BMI, body mass index; CMV, cytomegalovirus; ECMO, extracorporeal membrane oxygenation; eGFR, estimated glomerular filtration rate; FEV, forced expiratory volume; FVC, forced vital capacity; HCV, hepatitis C virus; HLA, human leukocyte antigen; LAS, lung allocation score; MAP, mean arterial pressure

© 2025 Sharma G et al. *JAMA Network Open.*

eTable 2: Baseline characteristics of patients in the study cohorts

Characteristics	Overall cohort (n= 51,933)	Training cohort (n=24,014)	Validation cohort (n=2,668)	Testing cohort (n=25,251)
Recipient-Level				
Recipient Age (Years)	59.00 [27.00, 71.00]	56 [25.00, 68.00]	56 [25.00, 68.00]	62.00[32.00,72.00]
Recipient Gender (Male)	29,905 (57.58)	13,224 (55.07)	1,517(56.86)	15,164 (60.05)
Ethnicity				
White	41,904 (80.70)	20,742 (86.40)	2,289 (85.79)	18,873 (74.74)
Black	4,435 (8.54)	1,787 (7.44)	202 (7.57)	2,446 (9.69)
Hispanic	4,067 (7.83)	1,102 (4.59)	127 (4.76)	2,838 (11.24)
Others	1,527 (2.94)	383 (1.59)	50 (1.87)	1,094 (4.33)
Body Mass Index (kg/m2)	25.25 [17.74, 32.64]	24.34 [17.21, 32.23]	24.33 [17.24, 32.32]	26.15[18.59,32.95]
Initial LAS Category				
<50	28,131 (54.17)	10,647 (44.34)	1,189 (44.57)	16,295 (64.54)
50-75	3,410 (6.57)	1,191 (4.96)	138 (5.17)	2,081 (8.24)
>75	2,367 (4.56)	649 (2.70)	58(2.17)	1,660(6.57)
NR	18,025 (34.71)	11,527 (48.00)	1,283 (48.09)	5215(20.65)
End LAS Category				
<50	24,575 (47.32)	9,954 (41.46)	1,138 (42.65)	13,513 (53.52)
50-75	5,117 (9.85)	2,027 (8.44)	219(8.21)	2,871 (11.37)
>75	4,611 (8.88)	1,437 (5.98)	134 (5.02)	3,040 (12.04)
NR	17,630 (33.95)	10,596 (44.11)	1,177 (44.12)	5,857 (23.20)
Initial Matched LAS Category				
<50	28,131 (54.17)	10,647 (44.34)	1,189 (44.57)	16,295 (64.53)
50-75	3,410 (6.57)	1,191 (4.96)	138 (5.17)	2,081 (8.24)
>75	2,367 (4.56)	649 (2.70)	58 (2.17)	1,660(6.57)
NR	18,025 (34.71)	11,527 (48.00)	1,283 (48.09)	5215 (20.65)
End Matched LAS Category				
<50	24,386 (46.96)	9,898 (41.23)	1,129 (42.32)	13,359 (52.90)
50-75	5,249 (10.11)	2,073 (8.63)	226 (8.47)	2,950(11.68)
>75	4,668 (8.99)	1,447 (6.03)	136 (5.10)	3,085 (12.12)
NR	17,630 (33.95)	10,596 (44.11)	1,177 (44.12)	5875 (23.20)
Functional Status at Registration				
Fully Active	3,994 (7.69)	3,468 (14.44)	392 (14.69)	134 (0.53)
Restricted activities/need assistance	32,712 (62.99)	14,883 (61.98)	1,651 (61.88)	16,178 (64.07)
Severe disabled	8,604 (16.57)	1,978 (8.23)	240 (9.00)	6,386 (25.29)
Completely disabled	3,297 (6.35)	1,030 (4.29)	98(3.67)	2169 (8.59)
NR/U/NA	3,326 (6.40)	2,655 (11.06)	287 (10.76)	384 (1.52)

© 2025 Sharma G et al. *JAMA Network Open.*

eTable 2: Baseline characteristics of patients in the study cohorts (contd..)

Characteristics	Overall cohort (n= 51,933)	Training cohort (n=24,014)	Validation cohort (n=2,668)	Testing cohort (n=25,251)
Functional Status at Transplant				
Fully Active	3,122 (6.01)	2,676 (11.14)	328 (12.29)	143 (0.57)
Restricted activities/need assistance	28,246 (54.39)	13,301 (55.39)	1,494 (56.00)	13,447 (53.27)
Severe disabled	11,027 (21.23)	3,199 (13.33)	340 (12.74)	7,473 (29.60)
Completely disabled	5,783 (11.14)	1,961 (8.17)	202 (7.57)	3,612 (14.31)
NR/U/NA	3,755 (7.23)	2,877 (11.98)	304 (11.39)	4,576 (18.12)
Chronic Steroid Use				
Yes	21,116 (40.66)	10,346 (43.08)	1,109 (41.57)	9,661 (38.26)
No	27,809(53.55)	11,136(46.37)	1,255(47.04)	15418(61.06)
U/NR	3,008(5.79)	2,532(10.54)	304(11.39)	172(0.68)
Life Support Ventilation (Yes)	2494(4.80)	1,108 (4.61)	98 (3.67)	1,288 (5.10)
CMV Status (Positive)	25,476 (49.06)	10,156 (42.29)	1,139 (42.69)	14,181 (56.16)
Negative	19,099(36.78)	7301(30.40)	813(30.47)	10985(43.50)
U/NR/ND	7,358(14.17)	6557(27.30)	716(26.84)	85(0.34)
Payment Source				
Private	24,776 (47.71)	12,819 (53.38)	1,451 (54.39)	10,506 (41.61)
Government/ Others/NR	27,157 (52.29)	11,195 (46.62)	1,217 (45.61)	14,745 (58.39)
HLA Mismatch Category				
Perfect/low mismatch	1,761 (3.39)	769 (3.20)	94 (3.52)	898 (3.56)
Moderate mismatch	17,521 (33.74)	7,907 (32.93)	855 (32.05)	8,759 (34.69)
High mismatch	27,275 (52.52)	11,974 (49.89)	1,340 (50.22)	13,961 (55.29)
NR	5,376 (10.35)	3,364 (14.00)	379 (14.21)	1,633 (6.47)
Diabetes (Yes)	8,347 (16.07)	3,115 (12.97)	349 (13.08)	4,883 (19.34)
N/U/NR	43,586(83.93)	20,899(87.03)	2319(86.92)	20,368(80.66)
Grouping Category				
A	17,380 (33.47)	10,246 (42.67)	1,144 (42.88)	5990(23.72)
B	2,472 (4.76)	1,155 (4.81)	131 (4.91)	1,186 (4.70)
C	4,946 (9.52)	3,148 (13.11)	351 (13.16)	1,447 (5.73)
D/NR	27,135 (52.25)	9,465 (39.41)	1,042 (39.06)	16,628 (65.85)
Pan-Bacterial Lung Infection				
Yes	1,142 (2.20)	585 (2.44)	61 (2.29)	496 (1.96)
No	46,502(89.54)	20,280(84.85)	2281(85.49)	23,941(94.81)
U/NR	4.289(8.26)	3,149(13.11)	326(12.22)	814(3.22)
Drug Therapy 2 Weeks Prior				
No	44,326 (85.35)	19,119 (79.62)	2,140(80.21)	23,067 (91.35)
Yes	4,589(8.84)	2,275(9.47)	236(8.85)	2,078 (8.23)
U/NR	3,018(5.81)	2,620(10.91)	292 (10.94)	106(0.42)
Cigarette Use				
No	16,763(32.28)	5,209 (21.69)	582(21.81)	10,972 (43.45)
Yes	23,556 (45.36)	8,358 (34.80)	925(34.67)	14,273 (56.52)

© 2025 Sharma G et al. *JAMA Network Open.*

eTable 2: Baseline characteristics of patients in the study cohorts (contd..)

Characteristics	Overall cohort (n= 51,933)	Training cohort (n=24,014)	Validation cohort (n=2,668)	Testing cohort (n=25,251)
NR	11,614(22.36)	10,447(43.50)	1,161(43.52)	6(0.02)
FEV	36.00 [14.00, 80.00] (n= 48758)	29.00 [13.00, 78.00] (n=22052)	30.00 [13.00, 78.00] (n=2457)	42.00 [16.00, 82.00] (n=24249)
Acute Rejection Episodes				
Yes	3,167 (6.10)	1,319 (5.49)	137 (5.13)	1,711 (6.78)
No	38,202(73.56)	13,222(55.06)	1,487(55.73)	23,493(93.04)
NR	10,564(20.34)	9,473(39.45)	1044(39.13)	47(0.19)
Multi-Organ Transplant (Yes)	320 (0.62)	66 (0.27)	8(0.30)	246 (0.97)
Inotropic Support with MAP				
Yes	2,255 (4.34)	690 (2.87)	76 (2.85)	1,489 (5.90)
FVC	48.00 [25.00, 84.00] (n=48755)	47.00 [25.00, 83.00] (n=22038)	46.50 [25.00, 83.00] (n=2456)	48.00 [25.00, 85.00] (n=24261)
Pre-Transplant Dialysis				
Yes	2,554 (4.92)	2,142 (8.92)	232 (8.70)	180 (0.71)
No	49,379(95.08)	21,872(91.08)	2,436(91.30)	25,071(99.29)
Prior Cardiac Surgery Before Listing				
Yes	1,920 (3.70)	616 (2.57)	71 (2.66)	1,233 (4.88)
U	10,681(20.57)	9588(39.93)	1064(39.88)	29(0.11)
N	39,332(75.74)	13810(57.51)	1533(57.46)	23,989(95.00)
Multiple Listing with Kidney (Yes)	132 (0.25)	20 (0.08)	1 (0.04)	111 (0.44)
No	51,801(99.75)	23,994(99.92)	2,667(99.96)	25,140(99.56)
Simultaneous Kidney Transplant (Yes)	119 (0.23)	18 (0.07)	1 (0.04)	100 (0.40)
No	51,814(99.77)	23,996(99.93)	2,667(99.96)	25,151(99.60)
Life Support at Transplant				
Yes	4,973(9.58)	22,278 (92.77)	2,511(94.12)	22,171(87.80)
N/NR	46,960 (90.42)	1,736(7.23)	157(5.88)	3,080 (12.20)
Ventilator Support since Listing(NO)	41,457 (79.83)	17,143 (71.39)	1,921 (72.00)	22,393 (88.68)
Yes	4,104 (7.90)	1,236 (5.15)	120 (4.50)	2,748 (10.88)
U	6,372 (12.27)	5,635(23.47)	627(23.50)	110 (0.44)
Total waiting list days	72.00 [3.00, 832.00]	137.00 [5.00, 993.00]	133.50 [5.00, 1000.00]	38.00 [3.00, 522.00]
HCV Serostatus				
Positive	1,066 (2.05)	376 (1.57)	31 (1.16)	659 (2.61)
Negative	47,579(91.62)	20,938(87.19)	2349(88.04)	24,292(96.20)
Not done/U/NR	3288(6.33)	2700(11.24)	288(10.79)	300(1.19)
Total Bilirubin	0.5[0.2,1.3]	0.5[0.2,1.4]	0.5[0.2,1.4]	0.4[0.2,1.2]
Diagnosis				
CF / Bronchiectasis	5,922(11.40)	3,644 (15.17)	413(15.48)	1,865 (7.39)

© 2025 Sharma G et al. *JAMA Network Open.*

eTable 2: Baseline characteristics of patients in the study cohorts (contd..)

Characteristics	Overall cohort (n= 51,933)	Training cohort (n=24,014)	Validation cohort (n=2,668)	Testing cohort (n=25,251)
COPD (incl. Alpha-1)	15,423 (29.70)	9,174(38.20)	1,007 (37.74)	5,242(20.76)
IPF / Fibrosis / ILD	20,25(38.99)	7,648(31.85)	823 (30.85)	11,780 (46.65)
Other	10,337(19.90)	3,548(14.77)	425(15.93)	6,364(25.20)
Creatinine	0.80 [0.50, 1.30]	0.80 [0.50, 1.30]	0.80 [0.50, 1.30]	0.80 [0.48, 1.30]
eGFR (50+) (mL/min/1.73m2)	93.16 [53.32, 127.72]	94.62 [53.19, 129.80]	95.07 [53.09, 129.33]	91.86 [53.45, 125.47]
ECMO Support (Yes)	2,051 (3.95)	99 (0.41)	10(0.37)	1,695 (6.71)
Donor Age (Years)	33.00 [16.00, 59.00]	30.00 [15.00, 57.00]	30.00 [15.00, 57.00]	35.00 [17.00, 60.00]
Donor Gender (Male)	31,724 (61.09)	14,757 (61.46)	1,659 (62.18)	15,308 (60.62)
Donor BMI (kg/m2)	25.00 [18.85, 36.13]	24.59 [18.61, 34.39]	24.62 [18.82, 34.09]	25.86[19.14,37.48]
Cause of Death				
Anoxia	11,427 (22.00)	2,448 (10.19)	272 (10.20)	8,707 (34.49)
Cerebrovascular / Stroke	15,855 (30.53)	8,259 (34.40)	909 (34.07)	6,687 (26.48)
Head Trauma	22,277 (42.90)	11,751 (48.94)	1,311 (49.14)	9,215 (36.49)
CNS tumor/Other/NR	2,374 (4.57)	1,556 (6.48)	176 (6.60)	642 (2.54)
Donor ethnicity				
Black	8,931 (17.20)	4,024 (16.76)	435 (16.30)	4,472 (17.71)
White	33,175(63.88)	16,211(67.51)	1787(66.98)	15177(60.10)
Hispanic/Latino	7955(15.32)	3,091(12.87)	371(13.91)	4493(17.79)
others/U	1872(3.60)	688(2.860	75(2.81)	1109(4.39)
Cigarette Use				
Yes	6,292 (12.12)	3,866(16.10)	445 (16.86)	1981(7.85)
No	42,806(82.43)	18,114(75.43)	2007(75.22)	22,685(89.84)
U/NR	2835(5.46)	2,034(8.47)	216(8.10)	585(2.32)
Diabetes (Donor) (Yes)	3,577 (6.89)	1,163 (4.84)	118(4.42)	2,296 (9.09)
No/U/NR	48,356(93.11)	22,851(95.16)	2550(95.58)	22,955(90.91)
Donor Cocaine History				
Hypertension (Donor) (Yes)	11,115 (21.40)	4,229 (17.61)	431 (16.15)	6,455(25.56)
(No/U/NR)	40,818(78.60)	19,785(82.39)	2,237(83.85)	18,799(74.44)
Cocaine Use History				
Yes	6,384 (12.29)	1,971 (8.21)	221 (8.28)	4,192 (16.60)
(No)	34,148(65.75)	16,203(67.47)	1789(67.05)	16156(63.98)
U/NR	11,401(21.95)	5840(24.32)	658(24.66)	4,903(19.42)
Heavy Alcohol Use (Donor)				
Yes	6,669 (12.84)	1,924 (8.01)	242 (9.07)	4,503 (17.83)
No	33,590(64.68)	12,267(51.08)	1329(49.81)	19,994(79.18)
U/NR	11,669(22.84)	9,823(40.91)	1097(41.12)	754(2.99)
Extended Criteria Donor (ECD)				
Yes	5,227 (10.06)	1,986 (8.27)	199 (7.46)	3,042 (12.05)
N/NR	46,706(89.94)	22,028(91.73)	2,469(92.54)	22,209(87.95)
Other Drug Use History				
Yes	17,793 (34.26)	6528 (27.18)	759 (28.25)	10,506 (41.61)
Ischemic time	5.19[2.63,10]	4.86[2.33,7.86]	4.93[2.33,7.79]	5.68[3.09,12.96]
Single or Double Lung Transplant (Double)	34,647 (66.70)	13,555 (56.45)	1,479 (55.43)	19,613 (77.67)
Single	17,286(33.30)	10,459(43.55)	1189(44.57)	5,638(22.33)

© 2025 Sharma G et al. *JAMA Network Open.*

eTable 2: Baseline characteristics of patients in the study cohorts (contd..)				
**Characteristics**	**Overall cohort (n= 51,933)**	**Training cohort (n=24,014)**	**Validation cohort (n=2,668)**	**Testing cohort (n=25,251)**
Distance of Donor Hospital to TX Center (Nautical Miles)	155.00 [0.00, 830.00]	109.00 [0.00, 553.00]	105.00 [0.00, 530.00]	195.00 [1.00, 926.00]
Transplant Year				
Before 1994	2,424 (4.67)	2,192 (9.13)	232 (8.70)	NA
1995–1999:	4,005 (7.71)	3,592 (14.96)	413 (15.48)	NA
2000–2004:	4,943 (9.52)	4,455 (18.55)	488(18.29)	NA
2005–2009:	6,793 (13.08)	6,111 (25.46)	682(25.56)	NA
2010–2014:	8,517 (16.40)	7,664 (31.91)	853 (31.97)	NA
2015–2019:	11,483 (22.11)	NA	NA	11,483(45.48)
2020–2025:	13,768 (26.51)	NA	NA	13768(54.52)
Length of Stay (Days)	17.00 [7.00, 78.00]	16.00 [6.00, 70.00]	15.00 [6.00, 68.00]	18.00 [8.00, 86.00]
Post-Transplant Ventilation Support				
No	1,369 (2.64)	806 (3.36)	87 (3.26)	476 (1.89)
<=48 hours	24,080 (46.37)	8,708 (36.26)	1004 (37.63)	14,368 (56.90)
>48 hours but < 5 days	6,997 (13.47)	2,230 (9.29)	229 (8.58)	4,538 (17.99)
>= 5 days	8,580 (16.52)	2,522 (10.50)	252 (9.45)	5,806 (22.99)
U/NR	10,907 (21.00)	9,748 (40.59)	1,096(41.08)	63 (0.25)
Creatinine				
Life Support (Yes)	4,973 (9.58)	1,736 (7.23)	188 (7.05)	2,049 (8.12)
No/NR	46,960 (90.42)	22,278 (92.77)	2,480 (92.95)	23,202 (91.88)
List Year				
1986–1999:	7,727 (14.88)	6,961 (28.99)	765 (28.67)	1(0.00)
2000–2009:	11,233 (21.62)	10,083 (41.99)	1,130 (42.35)	20(0.08)
2010–2019:	20,075 (38.65)	6,970 (29.02)	773 (28.97)	12,332(48.84)
2020–2025:	12,898 (24.84)			12,898(51.08)
Hemodynamic Systemic (mm/Hg)	38.00 [24.00, 79.00] (n= 45640)	38.00 [24.00, 79.00] (n=19149)	38.00 [24.00, 80.00] (n=2106)	39.00 [24.00, 79.00] (n=24093)
Hemodynamic Pulmonary Arterial Mean Pressure	25.00 [14.00, 50.00] (n= 45640)	25.00 [14.00, 50.00] (n=18176)	25.00 [14.00, 50.00] (n=1988)	25.00 [14.00, 50.00] (n=24385)
ECMO at transplant				
Yes	2051 (3.95)	329 (1.37)	27 (1.01)	1,695 (6.71)
No	49,882(96.05)	23,685(98.63)	2641(98.99)	23,556(93.29)
Transplant Region				
CT, ME, MA, NH, RI	1,605 (3.09)	703 (2.93)	70 (2.62)	832 (3.29)
DC, DE, MD, NJ, PA, WV	7,552 (14.54)	3,617 (15.06)	379 (14.21)	3,556 (14.08)
AL, AR, FL, GA, LA, MS, PR	5,146 (9.91)	2,398 (9.99)	246 (9.22)	2,502 (9.91)
OK, TX	5,582 (10.75)	2,477 (10.31)	260 (9.75)	2,845 (11.27)
AZ, CA, NV, NM, UT	8,025 (15.45)	3,328 (13.86)	393 (14.73)	4,304 (17.04)
AK, HI, ID, MT, OR, WA	1,318 (2.54)	700 (2.91)	83 (3.11)	535 (2.12)
IL, MN, ND, SD, WI	4,953 (9.54)	2,393 (9.97)	266 (9.97)	2,294 (9.08)
CO, IA, KS, MO, NE, WY	3,510 (6.76)	1,938 (8.07)	208 (7.80)	1,364 (5.40)
NY, VT	2,401 (4.62)	833 (3.47)	97 (3.64)	1,471 (5.83)
IN, MI, OH	6,174 (11.89)	2,709 (11.28)	335 (12.56)	3,130 (12.40)
KY, NC, SC, TN, VA	5,667 (10.91)	2,918 (12.15)	331 (12.41)	2,418 (9.58)

© 2025 Sharma G et al. *JAMA Network Open.*

eTable 2. Baseline Characteristics of Patients in the Study Cohorts. Data are presented as numbers (%) or median [5th, 95th percentile]. *Abbreviations:* BMI, body mass index; CF, cystic fibrosis; CMV, cytomegalovirus; COPD, chronic obstructive pulmonary disease; ECD, extended criteria donor; ECMO, extracorporeal membrane oxygenation; eGFR, estimated glomerular filtration rate; FEV, forced expiratory volume; FVC, forced vital capacity; HCV, hepatitis C virus; HLA, human leukocyte antigen; ILD, interstitial lung disease; LAS, lung allocation score; MAP, mean arterial pressure; N, No; NA, not applicable; NR, not reported; U, unknown.

© 2025 Sharma G et al. *JAMA Network Open.*

eTable 3. Clinical outcomes and follow-up duration by cohort			
**Outcome**	**Entire cohort (n=51,933)**	**Training cohort (n=24,014)**	**Testing cohort (n=25,251)**
Death / Retransplant (Events, %)	31,865 (61.36)	20,503 (85.38)	9,071 (35.92)
Time (Median, 95% CI)			
Follow-up, Year	8.97 (8.93–8.99)	16.94 (16.16–17.00)	4.01 (4.00–4.04)
Death or retransplant-free survival, Years	5.79 (5.71-5.88)	5.39 (5.26–5.50)	6.16 (6.05–6.28)

eTable 3. Clinical Outcomes and Follow-Up Duration by Cohorts. The primary outcome was a composite of death or retransplantation. *Abbreviation:* CI, confidence interval.

eTable 4. Univariable and multivariable cox regression analysis in the entire study cohort

Variables	UHR (95% CI)	p-value	AHR (95% CI)	p-value
Recipient Age (Years)	1.010 (1.010–1.011)	<0.001	1.008 (1.006-1.010)	<0.001
Recipient Gender (Male)	1.068 (1.045–1.092)	<0.001	1.060 (1.029-1.091)	<0.001
Ethnicity				
White	Ref		Ref	
Black	0.968 (0.929–1.008)	0.115	0.952 (0.907-0.999)	0.048
Hispanic	0.886 (0.844–0.930)	<0.001	0.889 (0.839-0.941)	<0.001
Others	0.887 (0.821–0.958)	0.002	0.914 (0.839-0.997)	0.043
Body Mass Index (kg/m2)	1.015 (1.012–1.017)	<0.001	1.003 (1.000-1.006)	0.025
Initial LAS Category				
<50	Ref		Ref	
50-75	1.117 (1.066–1.169)	<0.001	1.018 (0.957-1.083)	0.562
>75	1.123 (1.060–1.189)	<0.001	1.005 (0.918-1.100)	0.901
NR	1.181(1.153-1.211)	<0.001	1.043 (0.956-1.138)	0.340
End LAS Category				
<50	Ref		Ref	
50-75	1.095 (1.053–1.137)	<0.001	1.488 (1.128-1.964)	0.005
>75	1.098 (1.053–1.145)	<0.001	1.137 (0.674-1.917)	0.630
NR	1.202(1.172-1.233)	<0.001	1.006 (0.902-1.123)	0.902
Initial Matched LAS Category				
<50	Ref		NE	
50-75	1.117 (1.066–1.169)	<0.001	NE	NE
>75	1.123 (1.060–1.189)	<0.001	NE	NE
NR	1.181(1.153-1.211)	<0.001	NE	NE
End Matched LAS Category				
<50	Ref		Ref	
50-75	1.079 (1.037–1.120)	<0.001	0.709 (0.539-0.934)	0.014
>75	1.095 (1.050–1.141)	<0.001	0.856 (0.509-1.441)	0.56
NR	1.199(1.169-1.230)	<0.001		
Functional Status at Registration				
Fully Active	Ref		Ref	
Restricted activities/need assistance	0.987 (0.951 – 1.023)	0.473	1.1017 (1.0434 – 1.1632)	<0.001
Severe disabled	0.971 (0.928 – 1.017)	0.213	1.0512 (0.9807 – 1.1268)	0.158
Completely disabled	1.109 (1.046 – 1.175)	0.001	1.0878 (0.9952 – 1.1889)	0.064
NA / U / NR	1.245 (1.184 – 1.309)	<0.001	1.1300 (1.0293 – 1.2406)	0.010
Functional Status at Transplant				
Fully Active	Ref		Ref	
Restricted activities/need assistance	1.003(.963 - 1.045)	0.860	1.075 (1.014- 1.140)	0.014

© 2025 Sharma G et al. *JAMA Network Open.*

eTable 4. Univariable and multivariable cox regression analysis in the entire study cohort (contd..)

Variables	UHR (95% CI)	p-value	AHR (95% CI)	p-value
Severe disabled	1.028(0.981 - 1.076)	0.244	1.1648 (1.087 - 1.247)	<001
Completely disabled	1.178(1.118 - 1.240)	<001	1.307 (1.206 - 1.415)	<001
NR	1.312(1.245-1.382)	<001	1.195 (1.093- 1.305)	<001
Chronic Steroid Use(Yes)	1.108(1.083-1.134)	<0.001	1.045 (1.018-1.073)	0.001
Life Support Ventilation	1.143((1.086-1.204)	<0.001	1.078 (0.978-1.189)	0.129
CMV Status (Positive)	1.034(1.0092 – 1.0610)	0.007	0.971 (0.944-0.998)	0.040
Payment Source(Govt/Others/NR)				
Private	Ref		Ref	
Government/ Others/NR	1.147 (1.122–1.172)	<0.001	1.089(1.061-1.118)	<0.001
HLA Mismatch Category				
Perfect/low mismatch	Ref		Ref	
Moderate	1.073(1.006-1.144)	0.03	1.049 (0.975-1.128)	0.194
High	1.132(1.063-1.206)	<0.001	1.101 (1.025-1.183)	0.008
NR	0.187(0.108-0.271)	<0.001	1.127 (1.040-1.222)	0.004
Diabetes (Yes)	1.003(0.9727 – 1.0351)	0.830	1.063 (1.024-1.102)	0.001
Grouping Category				
A	Ref		Ref	
B	0.930 (0.881–0.982)	0.009	1.085 (0.987–1.193)	0.091
C	0.721 (0.694–0.750)	<0.001	1.088 (0.975–1.214)	0.130
D/NR	0.987 (0.964–1.011)	0.3	0.962 (0.895–1.033)	0.286
Pan-Bacterial Lung Infection (Yes)	0.879 (0.814-948)	0.001	1.174 (1.066-1.292)	0.001
Drug Therapy 2 Weeks Prior	0.947(0.912-0.985)	0.007	1.045 (0.989-1.103)	0.111
Cigarette Use (Yes)	1.167 (1.131–1.204)	<0.001	1.051 (1.017-1.088)	0.003
FEV	1.000(0.999-1.000)	0.654	1.000 (0.999-1.001)	0.654
Acute Rejection Episodes				
Yes	Ref		Ref	
No	0.789(0.754-0.826)	<001	0.890 (0.847-0.835)	<0.001
NR	0.975(0.929-1.023)	0.312	0.700 (0.553-0.908)	0.007
Multi-Organ Transplant (Yes)	0.889(0.749-1.054)	0.178	0.845 (0.657-1.087)	0.191
Inotropic Support with MAP (Yes)	1.034(0.976-1.094)	0.247	1.015 (0.955-1.079)	0.622
FVC	1.000(1.000-1.001)	0.020	0.999 (0.998-1.000)	0.576
Pre-Transplant Dialysis (Yes/Unknown)	1.341 (1.284-1.401)	<0.001	1.282 (1.111-1.471)	<0.001
Prior Cardiac Surgery	1.218 (1.144 – 1.298)	<0.001	1.218 (1.144-1.298)	<0.001
Multiple Listing with Kidney (Yes)	1.091(0.840-1.418)	0.514	0.784 (0.367-1.675)	0.53
Simultaneous Kidney Transplant (Yes)	1.138(0.867 – 1.494)	0.351	1.184 (0.520 – 2.694)	0.688

© 2025 Sharma G et al. *JAMA Network Open.*

eTable 4. Univariable and multivariable cox regression analysis in the entire study cohort (contd..)

Variables	UHR (95% CI)	p-value	AHR (95% CI)	p-value
Life Support at Transplant (Yes)	1.042 (1.000-1.085)	0.045	0.946 (0.877-1.021)	0.157
Ventilator Support since listing	1.068 (1.021-1.117)	<0.001	0.957 (0.894-1.024)	0.204
Listing Year				
2000–2009	0.863 (0.837–0.890)	<0.001	0.967 (0.881–1.060)	0.472
2010–2019	0.793 (0.769–0.817)	<0.001	0.991 (0.865–1.135)	0.894
2020–2025	0.725 (0.691–0.760)	<0.001	0.868 (0.720–1.046)	0.137
Total Days on Waiting List	0.999(0.999-0.999)	<0.001	0.999 (0.999-0.999)	0.003
HCV Serostatus (Positive)	1.023(0.9422 – 1.1119)	0.582	1.059 (0.966-1.161)	0.219
Total Bilirubin	1.021(1.015-1.026)	<0.001	1.017(1.010-1.024)	<0.001
Diagnosis				
CF / Bronchiectasis	Ref		Ref	
COPD (incl. Alpha-1)	1.412(1.361-1.465)	<0.001	1.053 (0.952-1.163)	0.315
IPF / Fibrosis / ILD	1.412 (1.361-1.465)	<0.001	1.064 (0.949-1.194)	0.289
Others	1.187(1.139-1.238)	<0.001	0.965 (0.865-1.077)	0.525
Creatinine (mg/dL)	1.048 (1.037-1.058)	<0.001	1.015 (0.993-1.035)	0.171
eGFR (50+) (mL/min/1.73m2)	0.996 (0.995–0.997)	<0.001	0.998 (0.997-0.999)	<0.001
ECMO at Transplant (Yes)	1.075(1.006-1.150)	0.232	1.032 (.921-1.155)	0.585
Donor Cocaine History (Yes)	1.037(1.001-1.074)	0.043	1.0571(1.014-1.101)	0.008
Donor Age (Years)	1.003 (1.002–1.004)	<0.001	1.004 (1.002-1.005)	<0.001
Donor Gender (Male)	1.022(0.999 – 1.046)	0.057	0.986 (0.957-1.016)	0.387
Donor BMI (kg/m2)	0.998 (0.996–1.000)	0.121	0.998 (0.996-1.001)	0.293
Cause of Death				
Anoxia	Ref		Ref	
Cerebrovascular / Stroke	1.159 (1.120–1.198)	<0.001	1.021 (0.980–1.065)	0.317
Head Trauma	1.084 (1.050–1.120)	<0.001	1.006 (0.969–1.045)	0.745
CNS Tumor/Other Specify/NR	1.204 (1.140–1.272)	<0.001	0.958 (0.886–1.037)	0.289
Donor ethnicity (black)	1.154(1.1210 – 1.1891)	<0.001	1.166 (1.127–1.207)	<0.001
Donor Cigarette Use (Yes)	1.167(1.1314 – 1.2050)	<0.001	1.060 (1.020-1.102)	0.003
Diabetes (Donor) (Yes)	1.088(1.0384 – 1.1403)	<0.001	1.084 (1.027–1.143)	0.003
Hypertension (Donor) (Yes)	1.078(1.048-1.108)	<0.001	0.976 (0.940-1.015)	0.235
Heavy Alcohol Use (Donor) (Yes)	1.002(0.996-1.040)	0.88	0.957 (0.919-0.997)	0.039
ECD Donor Status	1.144(1.102-1.188)	<0.001	1.068 (1.014-1.125)	0.013

© 2025 Sharma G et al. *JAMA Network Open.*

eTable 4. Univariable and multivariable cox regression analysis in the entire study cohort (contd..)

Variables	UHR (95% CI)	p-value	AHR (95% CI)	p-value
Other Drug Use History	0.987 (0.964-1.011)	0.294	1.030 (0.999-1.061)	0.053
Ischemic Time (hours)	0.962(0.957-0.968)	<0.001	1.002 (0.994-1.010)	0.528
Single or Double Lung Transplant (Single)	1.518(1.485-1.553)	<0.001	1.433 (1.388-1.481)	<0.001
Distance of Donor Hospital to TX Center (Nautical Miles)	1.000 (0.999–1.001)	0.109	0.999 (0.999-1.000)	0.305
Transplant Year				
Before 1994	Ref		Ref	
1995–1999	0.928 (0.880-0.978)	0.006	1.118 (0.995-1.256)	0.059
2000–2004	0 .798 (0.758-0.840)	<0.001	1.035 (0.874-1.226)	0.683
2005–2009	0.786 (0.748-0.826)	<0.001	1.107 (0.894-1.372)	0.350
2010–2014	0.747 (0.711-0.784)	<0.001	1.020 (0.806-1.290)	0.866
2015–2019	0.691 (0.658-0.727)	<0.001	0.938 (0.739-1.191)	0.603
2020–2025	0.668 (0.629-0.707)	<0.001	1.026 (0.787-1.339)	0.845
Length of Stay (Days)	1.001(1.000-1.002)	<0.001	1.002 (1.001-1.002)	<0.001
Post-Transplant Ventilator Support				
N	Ref		Ref	
<=48 hours	0.916 (0.854–0.983)	0.014	1.003 (0.926–1.086)	0.943
>48 hours but < 5 days	0.987 (0.916–1.065)	0.741	1.105 (1.015–1.204)	0.022
>= 5 days	1.436 (1.335–1.545)	<0.001	1.611 (1.480–1.754)	<0.001
Unknown / Not Reported	1.224 (1.141–1.314)	<0.001	1.029 (0.869–1.220)	0.738
Hemodynamic PA (Sys) at transplant (mm/Hg)	0.999 (0.998 – 0.999)	0.03	1.001 (0.999 – 1.003)	0.191
Hemodynamic Pulmonary Arterial Mean Pressure	0.998 (0.997 – 0.999)	0.002	0.997(0.994 – 1.000)	0.119
ECMO Pre-Transplant (Yes)	1.035(0.935-1.147)	0.497	0.963(0.809-1.147)	0.680
Transplant Region				
CT/ ME/ MA/ NH/ RI	Ref		Ref	
DC / DE / MD / NJ / PA / WV	1.124 (1.048–1.204)	0.001	1.067 (0.983–1.158)	0.122
AL / AR / FL / GA / LA / MS / PR	1.077 (1.001–1.158)	0.046	1.074 (0.986–1.169)	0.100
OK / TX	1.190 (1.108–1.278)	<0.001	1.158 (1.064–1.260)	0.001
AZ / CA / NV / NM / UT	1.089 (1.016–1.168)	0.016	1.181 (1.087–1.283)	<0.001
AK / HI / ID / MT / OR / WA	0.841 (0.765–0.924)	<0.001	0.983 (0.883–1.096)	0.762
IL / MN / ND / SD / WI	0.988 (0.919–1.063)	0.747	0.990 (0.910–1.078)	0.819
CO / IA / KS / MO / NE / WY	0.908 (0.842–0.980)	0.013	0.986 (0.903–1.076)	0.750
NY / VT	1.015 (0.932–1.106)	0.727	0.949 (0.858–1.051)	0.315
IN / MI / OH	1.010 (0.940–1.084)	0.789	0.995 (0.915–1.082)	0.905
KY / NC / SC / TN / VA	1.087 (1.013–1.168)	0.021	1.083 (0.996–1.179)	0.063

eTable 4. Univariable and Multivariable Cox Regression Analysis. The analysis included 51,933 patients in the study cohort. For continuous variables, HRs correspond to the change in hazard per one-unit increase (e.g., per 1 year for age, per 1 kg/m² for BMI, per 1 hour for ischemic time), where an HR = 1.0 indicates no change in hazard. For binary variables, HRs compare the coded category (value = 1) with its reference category (value = 0), as defined in the dataset; the reference category therefore has an HR = 1.0. For categorical variables with more than two levels, the reference group (indicated as Ref in the table) has an HR = 1.0, and HRs for other categories are relative to this group. *Abbreviations:* AHR, adjusted hazard ratio; CI, confidence interval; CMV, cytomegalovirus; COPD, chronic obstructive pulmonary disease; ECD, extended criteria donor; ECMO, extracorporeal membrane oxygenation; eGFR, estimated glomerular filtration rate; FEV, forced expiratory volume; FVC, forced vital capacity; HCV, hepatitis C virus; HLA, human leukocyte antigen; ILD, interstitial lung disease; LAS, lung allocation score; MAP, mean arterial pressure; N, No; NA, not applicable; NE, not estimable; NR, not reported; Ref, reference value i.e., 1; U, unknown; UHR, univariable hazard ratio; AHR, multivariable hazard ratio.

© 2025 Sharma G et al. *JAMA Network Open.*

eTable 5: Time-dependent model discrimination by transplant regions

Transplant Regions	n	$AUC_{(1yr)}$	LL $AUC_{(1yr)}$	UL $AUC_{(1yr)}$	$AUC_{(5yr)}$	LL $AUC_{(5yr)}$	UL $AUC_{(5yr)}$	$AUC_{(10yr)}$	LL $AUC_{(10yr)}$	UL $AUC_{(10yr)}$
1	832	0.77	0.73	0.82	0.68	0.63	0.73	NE	NE	NE
2	3556	0.64	0.61	0.67	0.65	0.62	0.67	0.70	0.39	0.97
3	2502	0.72	0.68	0.75	0.65	0.62	0.67	NE	NE	NE
4	2845	0.70	0.67	0.73	0.60	0.57	0.63	0.73	0.39	0.94
5	4304	0.70	0.67	0.73	0.64	0.62	0.67	0.64	0.41	0.89
6	535	0.68	0.60	0.75	0.61	0.54	0.68	0.38	0.27	0.50
7	2294	0.70	0.67	0.73	0.68	0.64	0.71	0.69	0.62	0.74
8	1364	0.71	0.66	0.76	0.60	0.56	0.64	0.56	0.32	0.76
9	1471	0.71	0.67	0.74	0.69	0.65	0.73	NE	NE	NE
10	3130	0.69	0.66	0.72	0.64	0.61	0.67	0.73	0.48	0.91
11	2418	0.71	0.67	0.74	0.69	0.65	0.71	0.89	0.72	0.99

eTable 5. Time-Dependent Model Discrimination by Transplant Regions. Data are Area Under the Receiver Operating Characteristic Curve (AUC) (95% CI) at 1-, 5-, and 10-years post-transplant. *Abbreviations:* AUC, Area Under the Curve; CI, confidence interval; LL, lower limit; NE, not estimable; UL, upper limit

© 2025 Sharma G et al. *JAMA Network Open.*

eTable 6. Overall model discrimination performance by transplant regions

Transplant Regions	n	C-stat	iAUC	LL iAUC	UL iAUC
1	832	0.66	0.64	0.62	0.71
2	3556	0.62	0.62	0.60	0.65
3	2502	0.63	0.64	0.62	0.67
4	2845	0.61	0.63	0.61	0.66
5	4304	0.63	0.65	0.63	0.67
6	535	0.61	0.62	0.56	0.69
7	2294	0.65	0.66	0.62	0.68
8	1364	0.61	0.61	0.58	0.66
9	1471	0.65	0.66	0.64	0.69
10	3130	0.63	0.63	0.60	0.64
11	2418	0.66	0.67	0.64	0.70

eTable 6. Overall Model Discrimination Performance by Transplant Regions.

Data are the C-statistic or integrated Area Under the Curve (iAUC) with 95% CI.

Abbreviations: CI, confidence interval; iAUC, integrated Area Under the Curve; LL, lower limit; UL, upper limit.

© 2025 Sharma G et al. *JAMA Network Open.*

eTable 7: Time-dependent model discrimination by age groups

Age Groups	n	$AUC_{(1yr)}$	LL $AUC_{(1yr)}$	UL $AUC_{(1yr)}$	$AUC_{(5yr)}$	LL $AUC_{(5yr)}$	UL $AUC_{(5yr)}$	$AUC_{(10yr)}$	LL $AUC_{(10yr)}$	UL $AUC_{(10yr)}$
<40 yrs	2254	0.74	0.70	0.78	0.63	0.60	0.66	0.60	0.39	0.74
40-60 yrs	8830	0.71	0.69	0.73	0.64	0.62	0.66	0.71	0.55	0.83
>60 yrs	14167	0.69	0.68	0.71	0.64	0.63	0.66	0.61	0.40	0.80

eTable 7. Time-Dependent Model Discrimination by Age Groups. Data are Area Under the Receiver Operating Characteristic Curve (AUC) (95% CI) at 1-, 5-, and 10-years post-transplant. *Abbreviations:* AUC, Area Under the Curve; CI, confidence interval; LL, lower limit; UL, upper limit

© 2025 Sharma G et al. *JAMA Network Open.*

eTable 8: Overall model discrimination performance by age groups					
Age groups	n	C-stat	iAUC	lower	upper
<40 yrs	2254	0.62	0.66	0.64	0.70
40-60 yrs	8830	0.64	0.66	0.65	0.68
>60 yrs	14167	0.64	0.64	0.62	0.66

eTable 8. Overall Model Discrimination Performance by Age Groups. Data are the C-statistic or integrated Area Under the Curve (iAUC) with 95% CI. *Abbreviations:* CI, confidence interval; iAUC, integrated Area Under the Curve.

© 2025 Sharma G et al. *JAMA Network Open.*

eTable 9: Comparison of risk prediction models for lung transplant outcomes						
**Study/Model**	**Primary Outcome**	**Key Predictors**	**Performance (AUC/C-Index)**	**Calibration plot/measure**	**Interpretability**	**Intended Use Case**
Current Study (Hybrid approach) (n=51,933)	1-, 5-, 10-yr death/re-Tx, Type: Time to event	9 clinical variables	iAUC: 0.61; C-index: 0.64	Yes	High (Point-based score)	Post-discharge long-term risk monitoring
Brahmbhatt *et al.* (LASSO, RF, CM) (n=16,964)[5]	1-yr survival, Type: Binary	~20 clinical variables	Poor for post-Tx survival (~0.55-0.62)	Yes	Moderate (Complex formula)	Pre-transplant organ allocation
Xia *et al.* (RF and other seven ML models) (n=802)[6]	Primary Graft Dysfunction within 72 hours, Type: Binary	9 clinical variables	AUC: 0.82	Yes	Low (Black box)	Early post-op complication prediction
Tian *et al.* (RSF, and cox regression) (n=504)[7]	Overall survival, Type: Time to an event	16 variables (ICU stay most important)	iAUC: 0.77	Yes (Only Brier score)	Low (Black box)	Post-transplant survival prediction
Yeo *et al.* (GBM) (n=29,364)[8]	1-yr mortality, Type: Binary	10 pre-Tx factors	AUC: 0.96	No	Low (Black box)	Pre-transplant 1-yr risk prediction
Zafar *et al.* (Cox-LASSO) (n=10,660)[9]	Long-term survival (1, 5, and 10 years), Type: Time to an event	Recipient, donor, Tx factors	C-index: 0.64-0.72	No	Moderate (Web tool)	Pre-transplant recipient-donor matching
Moro *et al.* (Survival Tree) (n=27,296)[10]	Overall survival, Type: Binary and Time to an event)	6 post-Tx variables	Not reported	No	High (Decision tree)	Post-transplant survival estimation

eTable 9. Comparison of Risk Prediction Models for Lung Transplant Outcomes. Abbreviations: CI, confidence interval; iAUC, integrated Area Under the Curve.

© 2025 Sharma G et al. *JAMA Network Open.*

eReferences

1. Riley RD, Snell KI, Ensor J, et al. Minimum sample size for developing a multivariable prediction model: PART II - binary and time-to-event outcomes. *Stat Med*. Mar 30 2019;38(7):1276-1296. doi:10.1002/sim.7992
2. Xie F, Chakraborty B, Ong MEH, Goldstein BA, Liu N. AutoScore: A Machine Learning-Based Automatic Clinical Score Generator and Its Application to Mortality Prediction Using Electronic Health Records. *JMIR Med Inform*. Oct 21 2020;8(10):e21798. doi:10.2196/21798
3. Xie, F., Ning, Y., Yuan, H., Goldstein, B. A., Ong, M. E. H., Liu, N., & Chakraborty, B. (2022). AutoScore-Survival: Developing interpretable machine learning-based time-toevent scores with right-censored survival data. Journal of Biomedical Informatics, 125, 103959. https://doi.org/10.1016/j.jbi.2021.103959
4. Binuya, M. A. E., Engelhardt, E. G., Schats, W., Schmidt, M. K., & Steyerberg, E. W. (2022). Methodological guidance for the evaluation and updating of clinical prediction models: A systematic review. BMC Medical Research Methodology, 22(1), 316. https://doi.org/10.1186/s12874-022-01801-8
5. Brahmbhatt JM, Hee Wai T, Goss CH, et al. The lung allocation score and other available models lack predictive accuracy for post-lung transplant survival. *J Heart Lung Transplant*. Aug 2022;41(8):1063-1074. doi:10.1016/j.healun.2022.05.008
6. Xia W, Liu W, He Z, et al. Machine Learning for Predicting Primary Graft Dysfunction After Lung Transplantation: An Interpretable Model Study. *Transplantation*. Jan 10 2025;(1534-6080 (Electronic))doi:10.1097/TP.0000000000005326
7. Tian D, Yan H-J, Huang H, et al. Machine Learning–Based Prognostic Model for Patients After Lung Transplantation. *JAMA Network Open*. 2023;6(5):e2312022-e2312022. doi:10.1001/jamanetworkopen.2023.12022
8. Yeo HJ, Noh D, Son E, Kwon S, Cho WH. Machine Learning for 1-Year Mortality Prediction in Lung Transplant Recipients: ISHLT Registry. *Transpl Int*. 2025;38(1432-2277 (Electronic)):14121. doi:10.3389/ti.2025.14121
9. Zafar F, Hossain MM, Zhang Y, et al. Lung Transplantation Advanced Prediction Tool: Determining Recipient's Outcome for a Certain Donor. *Transplantation*. Oct 1 2022;106(10):2019-2030. doi:10.1097/TP.0000000000004131
10. Moro A, Janjua HM, Rogers MP, et al. Survival Tree Provides Individualized Estimates of Survival After Lung Transplant. *J Surg Res*. Jul 2024;299(1095-8673 (Electronic)):195-204. doi:10.1016/j.jss.2024.04.017

© 2025 Sharma G et al. *JAMA Network Open.*
