# Supplementary material for: Development and Validation of a Hybrid Machine Learning Model to Predict Lung Transplant Outcomes
Source: JAMA Netw Open. 2025 Nov 25;8(11):e2545369. doi: 10.1001/jamanetworkopen.2025.45369 (PMC12648352; doi:10.1001/jamanetworkopen.2025.45369)
Supplement: Supplement 2. — Data Sharing Statement [file jamanetwopen-e2545369-s002.pdf]

Data Sharing Statement

Sharma. Development and Validation of a Hybrid Machine Learning Model to Predict Lung Transplant Outcomes. *JAMA Netw Open*. Published November 25, 2025.
doi:10.1001/jamanetworkopen.2025.45369

Data
Data available: No

Additional Information
Explanation for why data not available: Summary data and the web-based risk calculator (https://risk-calculator12.shinyapps.io/lung-transplant-risk/) are available. The underlying individual patient data from the UNOS-OPTN registry cannot be shared due to data use agreements and privacy regulations.
